# Supplementary material for: Predictive role of ctDNA in esophageal squamous cell carcinoma receiving definitive chemoradiotherapy combined with toripalimab
Source: Nat Commun. 2024 Mar 1;15:1919. doi: 10.1038/s41467-024-46307-7 (PMC10907344; doi:10.1038/s41467-024-46307-7)
Supplement: Supplementary file 1 — Supplementary file [file 41467_2024_46307_MOESM1_ESM.pdf]

**Supplementary file**


| Item | Page |
| --- | --- |
| **Supplementary methods：** Library preparation, sequencing and mutation detection | **2** |
| **Supplementary Table 1.** Patient baseline characteristics (N=42). | **4** |
| **Supplementary Table 2.** Univariate analyses of PFS and OS according to gene alterations in baseline ctDNA (N=40). | **6** |
| **Supplementary Table 3.** Univariate analyses of PFS and OS according to alteration of KEGG pathways in baseline ctDNA (N=40). | **7** |
| **Supplementary Table 4.** Comparison of the baseline ctDNA-positive rate based on clinical characteristics. | **8** |
| **Supplementary Table 5.** Univariate analyses of PFS and OS by maxVAF at baseline, during, and post-CRT | **9** |
| **Supplementary Figure 1.** NGS panel including 474 cancer-related genes. | **10** |
| **Supplementary Figure 2.** The distribution of variants forms between tumor and baseline plasm. | **11** |
| **Supplementary Figure 3.** VAF of the clone mutation in tumor tissue and baseline plasma. | **12** |
| **Supplementary Figure 4.** PFS and OS in baseline NFE2L2-wild type and NFE2L2-mutant patients. | **13** |
| **Supplementary Figure 5.** PFS and OS in baseline ctDNA-positive and ctDNA-negative patients. | **14** |
| **Supplementary Figure 6. P**athway analyses based on the baseline ctDNA | **15** |
| **Supplementary Figure 7.** PFS and OS of patients with higher or lower post-CRT bTMB (cut-off: 3 muts/mb). | **16** |
| **Supplementary Figure 8.** CR rate in TMB-high and TMB-low groups during (cut-off: 1 muts/mb) and post-CRT (cut-off: 3 muts/mb). | **17** |
| **Study Protocol** | **19** |

## Supplementary methods

### Library preparation, sequencing and mutation detection

Sequencing libraries were prepared using a KAPA Hyper Prep Kit (Roche, KAPA Biosystems). The capture reaction conducted using Dynabeads M-270 (Life Technologies), xGen Lockdown hybridisation, and a wash kit (Integrated DNA Technologies). The captured libraries were amplified using Illumina p5 (5-'AAT GAT ACG GCG ACC ACC GA 3') and p7 primers (5-'CAA GCA GAA GAC GGC ATA CGA GAT 3') in KAPA HiFi HotStart ReadyMix (KAPA Biosystems) and purified using Agencourt AMPure XP beads. Libraries were quantified by qPCR (KAPA Biosystems). A Bioanalyzer 2100 (Agilent Technologies) was used to determine the library fragment size. The target-enriched library was sequenced on a HiSeq4000 NGS platform (Illumina) according to the manufacturer's instructions. Quality-filtered reads were aligned to the reference human genome (build hg19) by applying the Burrows-Wheeler Aligner.

The criteria used for SNVs and indels filtration were as following: i) minimum ⩾3 variant supporting reads, ii) filtered if present in > 1% population frequency in the 1000 g or ExAC database, iii) filtered through an internally collected list of recurrent sequencing errors (⩾3 variant reads and ⩽20% variant allele frequency (VAF) in at least 30 of ~2000 normal samples) on the same sequencing platform. The final list of mutations was annotated using vcf2maf (variant effect predictor for annotation). Copy number variations were detected using FACETS43 to obtain tumor purity, ploidy, and clonal heterogeneity-adjusted copy number data. Fusion events were identified using the DELLY fusion calling tool to determine the number of chimeric reads (sequencing paired ends mapped to different genes) and split reads (spanning a fusion breakpoint) from the targeted DNA-seq data. Fusions were filtered if (i) split reads <3, paired reads <5, or (ii) lacked an intact kinase domain. All fusions were manually confirmed using the integrative genomics viewer. ctDNA variants that were not present in the corresponding primary tumor were rigorously evaluated and were considered true variants if the following criteria were met: (i) alter reads⩾3; (ii) absence in the internal database of clonal hematopoiesis variants obtained from peripheral blood leukocyte samples of approximately 500 healthy donors; (iii) variant allele frequency (VAF) = 0% in the paired peripheral blood leukocyte control sample, thereby excluding clonal hematopoiesis variants or germline mutations.

### Diagnostic and follow up procedure

All patients were staged or assessed for response using PET/CT, CT, and esophagoscopy at the time of diagnosis and three months after completing radiation. During the follow-up period, patients were scheduled for regular visits every 9 weeks in the first year, every 3 months in the second year, and then every 6 months thereafter.

The follow-up included physical examinations, serologic tests, esophageal barium X-ray, and enhanced CT scans of the chest and abdomen. During the first three years, esophagoscopy was performed every six months and pathologic biopsies were taken if suspicious lesions were found. PET-CT scans were required for uncertain or metastatic lesions that could not be determined during regular follow-up.

Tumor response was assessed according to the Response Evaluation Criteria in Solid Tumors (RECIST; version 1.1). Complete response (CR) was defined based on comprehensive methods and the following criteria: 1) absence of any identifiable lesion, budding, or ulceration on esophagogastroduodenoscopy (EGD); 2) negative endoscopic findings confirmed by bite-on-bite biopsy; 3) no evidence of local or distant recurrences, and a maximum standardized uptake value (SUVmax) in the primary region at a normal physiological level or distributed in an esophagitis pattern according to PET imaging. If there was any uncertainty in the results, a reassessment was performed within 6 weeks to determine the final response.

**Supplementary Table 1.** Patient baseline characteristics (N=42).

| Characteristic | No. (%) |
|---|---|
| Age (years) | |
| Median (IQR) | 56 (53–63) |
| Sex | |
| Male | 32 (76) |
| Female | 10 (24) |
| Smoking history | |
| Yes | 22 (52) |
| No | 20 (48) |
| Alcohol history | |
| Yes | 18 (43) |
| No | 24 (57) |
| ECOG performance status | |
| 0 | 31 (74) |
| 1–2 | 11 (26) |
| Tumor location | |
| Upper | 21 (50) |
| Middle | 15 (36) |
| Distal | 6 (14) |
| Primary tumor length (cm) | |
| Median (IQR) | 5 (4–6) |
| ≤5 | 26 (62) |
| >5 | 16 (38) |
| Clinical T stage | |
| T1 | 2 (5) |

| | |
|---|---|
| T2 | 8 (19) |
| T3 | 23 (55) |
| T4 | 9 (21) |
| Clinical N stage | |
| N1 | 8 (19) |
| N2 | 23 (55) |
| N3 | 11 (26) |
| Clinical TNM stage | |
| I-III | 26 (62) |
| IVA | 16 (38) |
| PD-L1 | |
| CPS≥10 | 11 (28) |
| CPS＜10 | 29 (72) |

Abbreviations: IQR, interquartile range; ECOG, Eastern Cooperative Oncology Group; CPS, Combined Positive Score;

**Supplementary Table 2.** Univariate analyses of PFS and OS according to gene alterations in baseline ctDNA (N=40).

| Gene | Mutant Frequency (N) | PFS | | OS | |
|---|---|---|---|---|---|
| | | HR (95% CI) | P-value | HR (95% CI) | P-value |
| *NFE2L2* (MUT vs. WT) | 6 | 3.33 (1.28-8.63) | 0.009 | 3.93 (1.47-10.50) | 0.003 |
| *TP53* (MUT vs. WT) | 27 | 1.25 (0.56-2.78) | 0.586 | 1.41 (0.54-3.67) | 0.482 |
| *CDKN2A* (MUT vs. WT) | 8 | 0.54 (0.19-1.56) | 0.248 | 0.42 (0.10-1.83) | 0.235 |
| *LRP1B* (MUT vs. WT) | 5 | 1.69 (0.58-4.91) | 0.327 | 1.48 (0.43-5.06) | 0.529 |
| *NOTCH1* (MUT vs. WT) | 4 | 1.19 (0.36-3.95) | 0.778 | 1.13 (0.26-4.88) | 0.869 |
| *EP300* (MUT vs. WT) | 3 | 1.38 (0.33-5.84) | 0.661 | 1.79 (0.41-7.75) | 0.428 |
| *ASXL1* (MUT vs. WT) | 3 | 1.26 (0.30-5.37) | 0.750 | 1.08 (0.25-4.67) | 0.917 |
| *FLT1* (MUT vs. WT) | 3 | 1.00 (0.24-4.25) | 0.997 | 1.36 (0.32-5.89) | 0.679 |
| *CBL* (MUT vs. WT) | 3 | 0.00 (0.00-Inf) | 0.063 | 0.00 (0.00-Inf) | 0.146 |
| *DNMT3A* (MUT vs. WT) | 3 | 0.00 (0.00-Inf) | 0.063 | 0.00 (0.00-Inf) | 0.146 |

Abbreviations: HR, hazard ratios; 95%CI, 95%-confidence intervals; OS, overall survival; PFS, progression-free survival; MUT, mutation; WT, wild type. *Only the gene alteration with frequency ≥ 3 was included. P value was determined by the univariate cox proportional hazards model.

**Supplementary Table 3.** Univariate analyses of PFS and OS according to alteration of pathways in baseline ctDNA (N=40).

| Pathways | PFS | | OS | |
|---|---|---|---|---|
| | HR (95% CI) | P-value | HR (95% CI) | P-value |
| NFE2L2 | 3.24(1.30-8.08) | 0.008 | 3.85(1.50-9.91) | 0.003 |
| Cell cycle | 0.55(0.21-1.46) | 0.222 | 0.30(0.07-1.28) | 0.084 |
| HIPPO | 0.49(0.07-3.63) | 0.477 | 1.37E-08(0-Inf) | 0.243 |
| NOTCH | 1.23(0.49-3.04) | 0.661 | 1.41(0.51-3.88) | 0.507 |
| PI3K | 1.42(0.60-3.37) | 0.423 | 1.11(0.40-3.05) | 0.844 |
| RTK | 1.32(0.60-2.89) | 0.486 | 1.08(0.43-2.72) | 0.862 |
| TGF | 0.46(0.06-3.42) | 0.439 | 0.64(0.09-4.82) | 0.666 |
| TP53 | 1.25(0.56-2.78) | 0.586 | 1.41(0.54-3.67) | 0.482 |
| Antimicrobials | 0.87(0.35-2.15) | 0.757 | 0.48(0.14-1.63) | 0.226 |
| BCR signaling pathway | 1.86(0.64-5.42) | 0.249 | 1.47(0.43-5.02) | 0.538 |
| TCR signaling pathway | 0.69(0.26-1.83) | 0.458 | 0.48(0.14-1.66) | 0.238 |
| Natural killer cell cytotoxicity | 1.21(0.46-3.19) | 0.704 | 0.86(0.25-2.92) | 0.803 |
| Cytokine receptors | 1.72(0.59-5.02) | 0.319 | 1.93(0.64-5.78) | 0.233 |
| Cytokines | 0.79(0.24-2.63) | 0.702 | 0.57(0.13-2.47) | 0.447 |

Abbreviations: HR, hazard ratios; 95%CI, 95%-confidence intervals; OS, overall survival; PFS, progression-free survival; P value was determined by the univariate cox proportional hazards model.

**Supplementary Table 4.** Comparison of the baseline ctDNA-positive rate based on clinical characteristics

| Characteristic | ctDNA-positive (n = 29), % | ctDNA-negative (n = 11), % | *P*-value |
|---|---|---|---|
| Age (years) | | | 0.87 |
| ≤56 | 15 (51.7) | 6 (54.5) | |
| >56 | 14 (48.3) | 5 (45.5) | |
| Sex | | | 0.84 |
| Male | 7 (24.1) | 3 (27.3) | |
| Female | 22 (75.9) | 8 (72.7) | |
| ECOG performance status | | | 0.69 |
| 0 | 20 (69.0) | 9 (81.8) | |
| 1–2 | 9 (31.0) | 2 (18.2) | |
| Smoking history | | | 0.38 |
| No | 15 (51.7) | 4 (36.4) | |
| Yes | 14 (48.3) | 7 (63.6) | |
| Drinking history | | | 0.72 |
| No | 15 (51.7) | 7 (63.6) | |
| Yes | 14 (48.3) | 4 (36.4) | |
| Tumor location (Thoracic) | | | 0.39 |
| Upper | 13 (44.8) | 7 (63.6) | |
| Middle | 12 (41.4) | 2 (18.2) | |
| Lower | 4 (13.8) | 2 (18.2) | |
| Tumor length | | | **0.023** |
| ≤5 cm | 15 (51.7) | 10 (90.9) | |
| >5 cm | 14 (48.3) | 1 (9.1) | |
| Clinical TNM stage | | | **0.036** |
| I-III | 16 (55.2) | 10 (90.9) | |
| IVA | 13 (44.8) | 1 (9.1) | |

Abbreviations: ECOG, Eastern Cooperative Oncology Group. P value was determined by chi-square test expect for tumour location which was determined by two-tailed Fisher’s exact test.

**Supplementary Table 5.** Univariate analyses of PFS and OS by maxVAF at baseline, during, and post-CRT

| PFS | | | OS | | |
|---|---|---|---|---|---|
| maxVAF | HR (95% CI) | *P*-value | maxVAF | HR (95% CI) | *P*-value |
| Baseline | 1.04 (0.94-1.16) | 0.47 | Baseline | 1.03 (0.93-1.14) | 0.54 |
| During-CRT | 1.45 (0.98-2.14) | 0.067 | During-CRT | 1.20 (0.79-1.81) | 0.39 |
| Post-CRT | 1.94 (1.16-3.27) | 0.011 | Post-CRT | 1.50 (0.93-2.41) | 0.095 |

Abbreviations: OS, overall survival; PFS, progression-free survival; maxVAF, maximal variant allele frequency; CRT, chemoradiotherapy. P value was determined by the univariate cox proportional hazards model.

**Supplementary Figure 1.** NGS panel including 474 cancer-related genes.

| Genes list ( 474 ) | | | | | | | | | | | |
|---|---|---|---|---|---|---|---|---|---|---|---|
| ABCB1(MDR1) | ABCB4 | ABCC2(MRP2) | ABL1 | ADGRB3(BAI3) | ADH1B | AFDN(MLLT4) | AIP | AKT1 | AKT2 | AKT3 | ALDH2 |
| ALK | AMER1(FAM123B) | APC | APEX1 | AR | ARAF | ARID1A | ARID1B | ARID2 | ARID5B | ASCL4 | ASXL1 |
| ATF1 | ATIC | ATM | ATR | ATRX | AURKA | AURKB | AXIN2 | AXL | B2M | BAD | BAK1 |
| BAP1 | BARD1 | BAX | BCL2 | BCL2L1(BCL-XL) | BCL2L11(BIM) | BCL3 | BCR | BIRC3 | BLM | BMPR1A | BRAF |
| BRCA1 | BRCA2 | BRD4 | BRIP1 | BTG2 | BTK | BUB1B | CASP8 | CBL | CBLB | CCN6(WISP3) | CCND1 |
| CCNE1 | CD274(PD-L1) | CD74 | CDA | CDC73 | CDH1 | CDK10 | CDK12 | CDK4 | CDK6 | CDK8 | CDKN1A |
| CDKN1B | CDKN1C | CDKN2A | CDKN2B | CDKN2C | CEBPA | CEP57 | CHD4 | CHD8 | CHEK1 | CHEK2 | CREBBP |
| CRKL | CSF1R | CTCF | CTLA4 | CTNNB1 | CUL3 | CUX1 | CXCL8 (IL8) | CXCR4 | CYLD | CYP19A1 | CYP2A13 |
| CYP2A6 | CYP2A7 | CYP2B6 | CYP2C19 | CYP2C9 | CYP2D6 | CYP3A4 | CYP3A5 | CYSLTR2 | DAXX | DDR2 | DENND1A |
| DHFR | DICER1 | DLL3 | DNMT3A | DOT1L | DPYD | DTL(CDT2) | DUSP2 | EGFR | EIF1AX | EMSY(c11orf30) | EP300 |
| EPAS1 | EPCAM | EPHA2 | EPHA3 | EPHA5 | ERBB2(HER2) | ERBB3 | ERBB4 | ERBIN(ERBB2IP) | ERCC1 | ERCC2 | ERCC3 |
| ERCC4 | ERCC5 | ESR1 | ETV1 | ETV4 | ETV5 | ETV6 | EWSR1 | EXT1 | EXT2 | EZH2 | EZR |
| FANCA | FANCC | FANCD2 | FANCE | FANCF | FANCG | FANCI | FANCL | FANCM | FAT1 | FBXW7 | FGF19 |
| FGFR1 | FGFR2 | FGFR3 | FGFR4 | FH | FLCN | FLT1(VEGFR1) | FLT3 | FLT4 | FOXA1 | FOXL2 | FOXO3(FOXO3A) |
| FOXP1 | FRG1 | GATA1 | GATA2 | GATA3 | GATA4 | GATA6 | GNA11 | GNAQ | GNAS | GRIN2A | GRM3 |
| GRM8 | GSTM1 | GSTM4 | GSTP1 | GSTT1 | HDAC1 | HDAC2 | HDAC9 | HGF | HLA-A | HMOX1 | HNF1A |
| HNF1B | HRAS | HSPB1 | IDH1 | IDH2 | IFNA6 | IFNB1 | IFNE | IFNG | IFNGR1 | IFNGR2 | IGF1R |
| IGF2 | IKBKE | IKZF1 | IL13 | IL1A | IL7R | INPP4B | IRF1 | IRF2 | ITGB6 | JAK1 | JAK2 |
| JAK3 | JARID2 | JUN | KDM5A | KDR(VEGFR2) | KEAP1 | KIF1B | KIT | KITLG | KLLN | KMT2A(MLL) | KMT2B |
| KMT2C | KMT2D(MLL2) | KRAS | LHCGR | LIG3 | LIG4 | LIN28B | LMO1 | LRP1B | LYN | LZTR1 | MALT1 |
| MAPK3(ERK1) | MAX | MCL1 | MDM2 | MDM4 | MECOM | MAP2K1(MEK1) | MAP2K2(MEK2) | MAP2K4 | MAP3K1 | MAP3K4 | MAPK1(ERK2) |
| MITF | MLH1 | MLH3 | MLLT1 | MLLT3 | MMP1 | MED12 | MEF2B | MEN1 | MET | MGMT | MIF |
| MTRR | MUC5B | MUTYH | MYC | MYCL(MYCL1) | MYCN | MPL | MRE11(MRE11A) | MSH2 | MSH6 | MTHFR | MTOR |
| NF1 | NF2 | NFE2L2 | NFKB1 | NFKBIA | NKX2-1 | MYD88 | MYH9 | NAT1 | NBN | NCOR1 | NEIL1 |
| NQO1 | NRAS | NRG1 | NSD1 | NTRK1 | NTRK2 | NOS2 | NOS3 | NOTCH1 | NOTCH2 | NOTCH3 | NPM1 |
| PARP1 | PARP2 | PAX5 | PBRM1 | PDCD1(PD1) | PDCD1LG2(PD-L2) | NTRK3 | NUTM1 | PAK2 | PAK3 | PALB2 | PALLD |
| PIK3C3 | PIK3CA | PIK3CD | PIK3R1 | PIK3R2 | PKHD1 | PDE11A | PDGFRA | PDGFRB | PDK1 | PGR | PHOX2B |
| PNKP(PNK) | POLD1 | POLD3 | POLE | POLH | POT1 | PLAG1 | PLCB4 | PLK1 | PMAIP1(NOXA) | PMS1 | PMS2 |
| PRKCB | PRKCI | PRKDC | PRKN(PARK2) | PRSS1 | PRSS3 | PPARD | PRDM1 | PREX2 | PRF1 | PRKACA | PRKAR1A |
| RAC1 | RAC3 | RAD50 | RAD51 | RAD51B | RAD51C | PTCH1 | PTEN | PTK2 | PTPN11 | PTPN13 | QKI |
| RASGEF1A | RB1 | RCC1 | RECQL4 | RELA | RELN | RAD51D | RAD54L | RAD9A | RAF1 | RARA | RARG |
| RPTOR | RRM1 | RUNX1 | RUNX1T1 | SBDS | SDC4 | RET | | RHOA | RICTOR | RNF43 | ROS1 |
| SETBP1 | SETD2 | SF3B1 | SGK1 | SKP2 | SLC34A2 | SDHA | SDHB | SDHC | SDHD | SEPTIN9(SEPT9) | SERPINE1(PAI1) |
| SMARCB1 | SMO | SOCS1 | SOS1 | SOX2 | SPOP | SLC3A2 | SMAD2 | SMAD3 | SMAD4 | SMAD7 | SMARCA4 |
| STAT1 | STAT3 | STK11 | STMN1 | SUFU | SUMO1 | SPRED1 | SPRY4 | SRC | SRSF2 | SRY | STAG2 |
| TERC | TERT | TET2 | TFG | TGFB1(TGFβ) | TGFBR2 | TACC3 | TAP1 | TAP2 | TBK1 | TEK | TEKT4 |
| TNFRSF11A | TNFRSF14 | TNFRSF19 | TNFRSF1B | TNFSF11 | TOP1 | THADA | TMEM127 | TMEM167A | TMPRSS2 | TNF (TNFa) | TNFAIP3 |
| TSHR | TTF1 | TUBB3 | TYMS | U2AF1 | UGT1A1 | TOP2A | TP53 | TP63 | TPMT | TSC1 | TSC2 |
| UNG | VAMP2 | VEGFA | VHL | WAS | WRN | WT1 | XPA | XPC | XRCC1 | XRCC2 | XRCC3 |
| XRCC4 | XRCC5 | YAP1 | ZNF2 | ZNF217 | ZNF703 | | | | | | |

**Supplementary Figure 2.** The distribution of variants forms between tumor and baseline plasm.

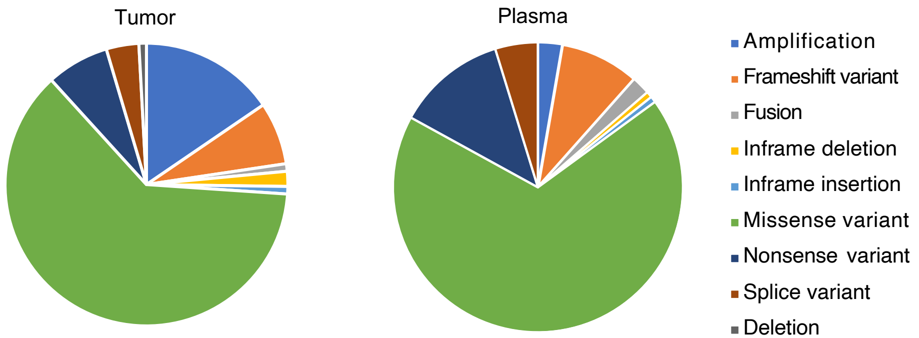

**Supplementary Figure 3.** VAF of the clone mutation in tumor tissue and baseline plasma. **(A)** correlation of VAF of the clone mutation in tumor tissue and the corresponding baseline plasma. **(B)** Comparison of the AF of shared and unique variants in tumor and corresponding baseline plasma. Data are presented as mean values +/- SD. P-values (p) were determined by two-tailed unpaired t-tests.

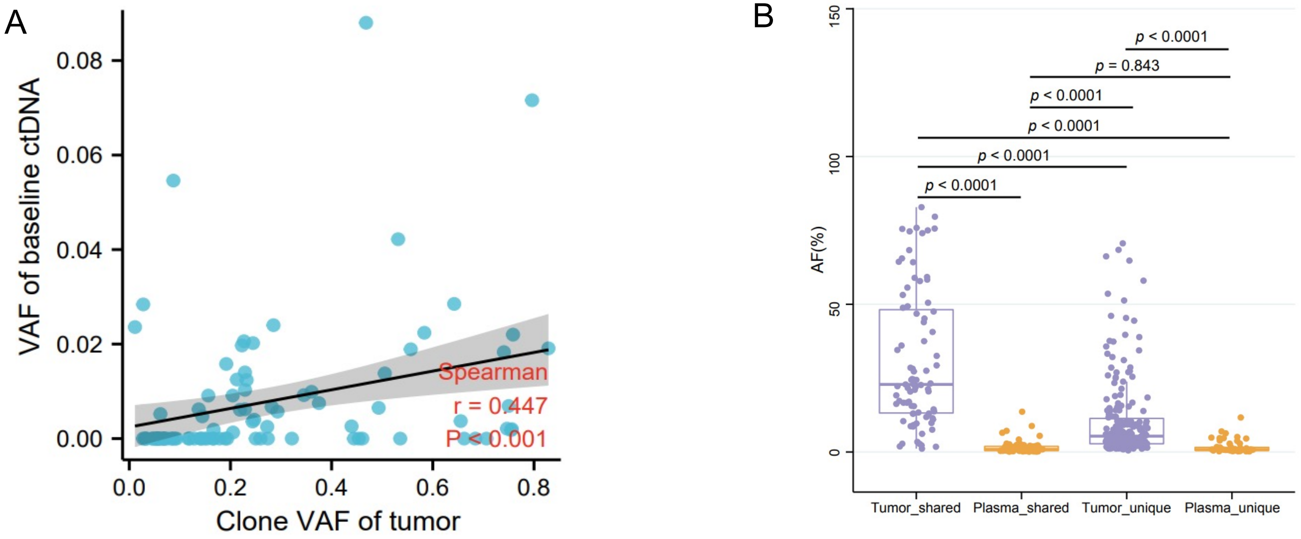

**Supplementary Figure 4.** PFS and OS in baseline *NFE2L2*-wild type and *NFE2L2*-mutant patients. P-values (p) were determined by Log Rank Test.

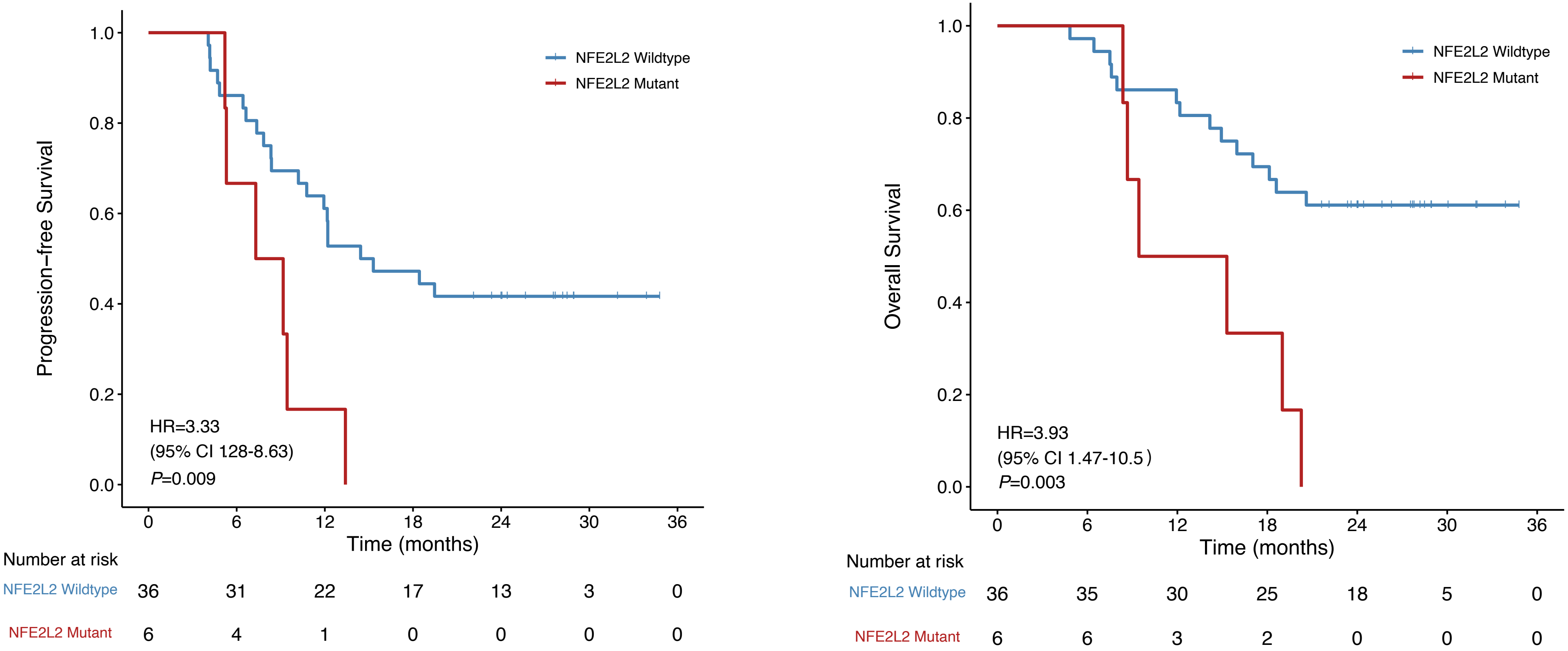

**Supplementary Figure 5.** Pathway analyses based on the baseline ctDNA.(n=40)

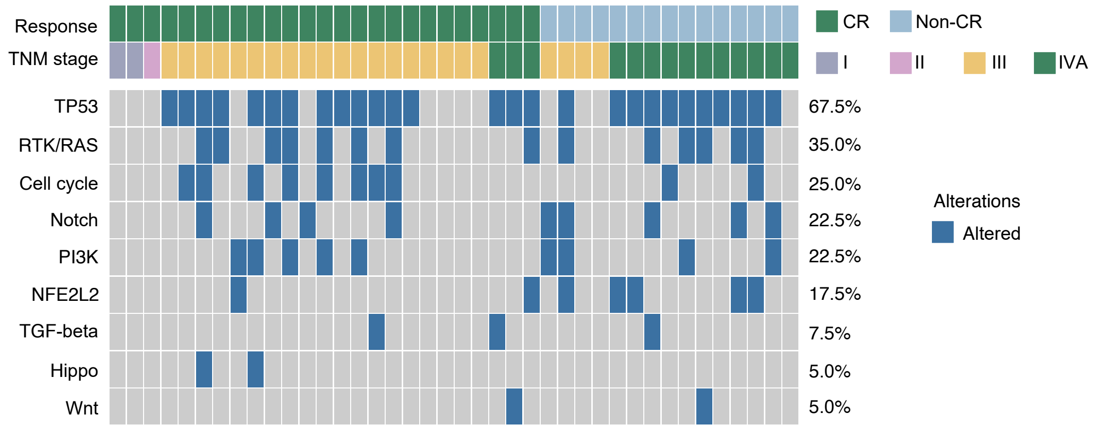

**Supplementary Figure 6.** PFS and OS in baseline ctDNA-positive and ctDNA-negative patients. P-values (p) were determined by Log Rank Test.

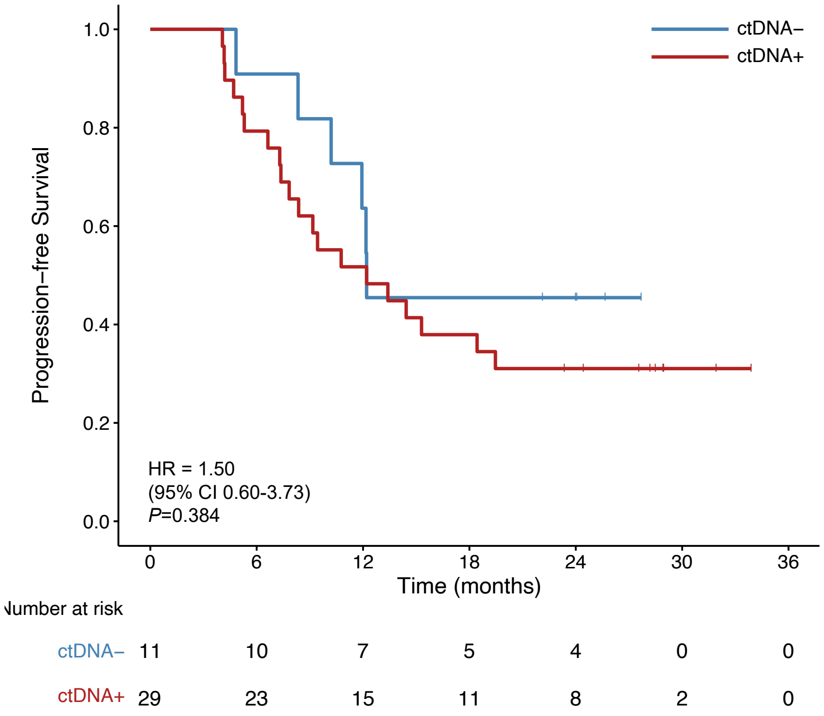


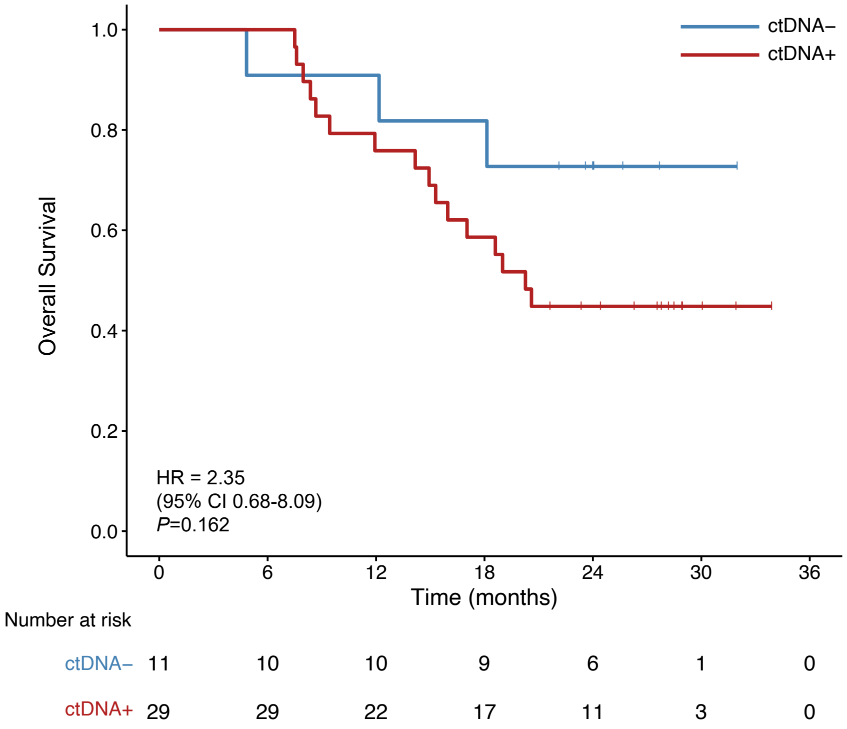

**Supplementary Figure 7.** PFS and OS of patients with higher or lower post-CRT bTMB (cut-off: 3 muts/mb). P-values (p) were determined by Log Rank Test.

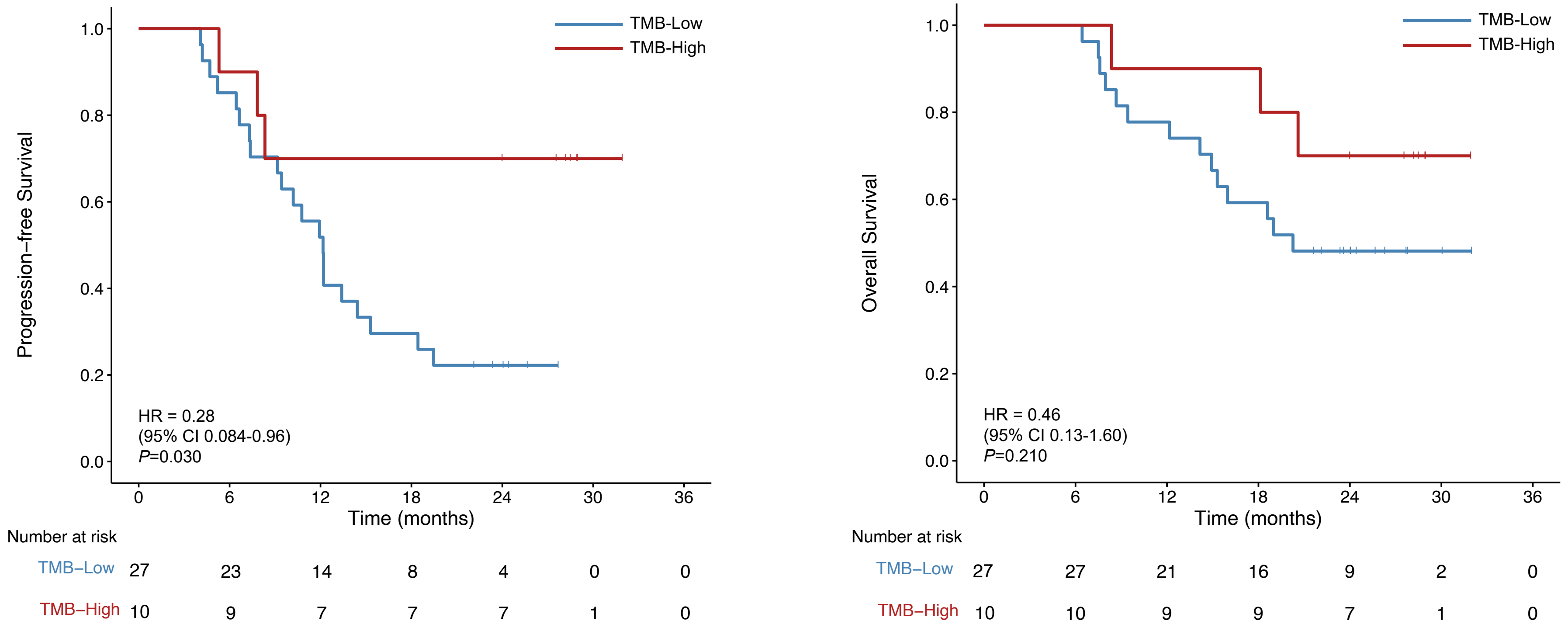

**Supplementary Figure 8.** CR rate in TMB-high and TMB-low groups during (cut-off: 1 muts/mb) and post-CRT (cut-off: 3 muts/mb). P-values (p) were determined by two-tailed Fisher's exact test;

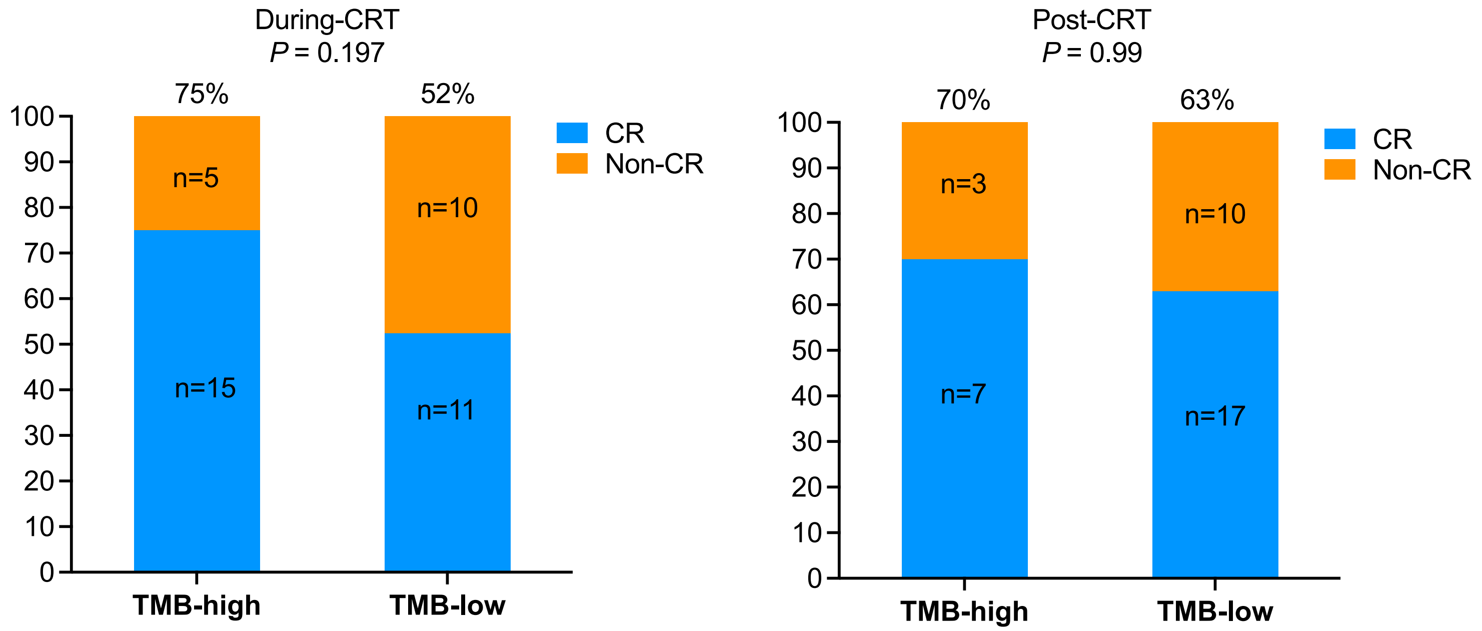

# A PHASE II TRIAL OF COMBINATION OF TORIPALIMAB AND DEFINITIVE CHEMORADIOTHERAPY IN ESOPHAGEAL SQUAMOUS CELL CARCINOMA

# (EC-CRT-001)

## Clinical Research Protocol

## Principal Investigator

Professor Mengzhong LIU
Department of Radiation Oncology, Sun Yat-sen University Cancer Center,
No.651 Dongfeng East Road, Guangzhou 510060,China.
E-mail: liumz@sysucc.org.cn

Professor Yonghong HU
Department of Radiation Oncology, Sun Yat-sen University Cancer Center,
No.651 Dongfeng East Road, Guangzhou 510060,China.
E-mail: huyh@sysucc.org.cn

Professor Mian XI
Department of Radiation Oncology, Sun Yat-sen University Cancer Center,
No.651 Dongfeng East Road, Guangzhou 510060,China.
E-mail: ximian@sysucc.org.cn

## Participating center: Sun Yat-sen University Cancer Center

## Version number / date: 1.0 / 20rd March 2019

# Table of Contents

1. Schema.....3

2. Summary .....6

3. Background.....7

4. Objective .....9

5. Plan .....9

6. Route.....27

7. References.....28

# 1. Schema

| | |
|---|---|
| **Research Topic** | A Phase II Trial of Combination of Toripalimab and Definitive Chemoradiotherapy in Esophageal Squamous Cell Carcinoma. |
| **Research Purpose** | To evaluate the efficacy, safety, and biomarkers of the combination of toripalimab (an anti-PD-1 antibody) and definitive chemoradiotherapy (CRT) in locally advanced esophageal squamous cell carcinoma (ESCC). |
| **Research Design** | Prospective, single-center, single-arm, phase II trial |
| **Principal Investigator** | Dr. Mengzhong Liu, Yonghong Hu, and Mian Xi |
| **Research Object** | Unresectable locally advanced esophageal squamous cell carcinoma |
| **Research Endpoints** | 1) Primary endpoints: clinical complete response (cCR)<br>2) Secondary endpoints: progression-free survival (PFS), overall survival (OS), duration of response (DOR), toxicity, and quality of life.<br>3) Exploratory endpoints: To investigate the impact of programmed cell death-ligand 1 (PD-L1) expression, immune cell infiltration in the tumor microenvironment, tumor mutational burden, genetic biomarkers, and dynamic ctDNA on clinical response and survival. |
| **Inclusion Criteria** | 1) Histologically confirmed squamous cell carcinoma of the esophagus;<br>2) Not suitable for surgery (either for medical reasons or patient's choice);<br>3) No prior cancer therapy;<br>4) Estimated life expectancy >6 months;<br>5) Age at diagnosis being 18-70 years;<br>6) The function of important organs meets the following requirements:a.white blood cell count (WBC) ≥ $4.0\times10^9$/L, absolute neutrophil count (ANC) ≥ $1.5\times10^9$/L; b. platelets ≥ $100\times10^9$/L; c. hemoglobin ≥ 9g/dL; d. serum albumin ≥ 2.8g/dL; e. total bilirubin ≤ 1.5×ULN, ALT, AST and/or AKP ≤ 2.5×ULN; f. serum creatinine ≤ 1.5×ULN or creatinine clearance rate >60 mL/min;<br>7) PS score ≤2;<br>8) Ability to understand the study and sign informed consent. |

| | |
|---|---|
| **Exclusion Criteria** | 1) Patients who have been treated previously with anti-tumor therapy (including chemotherapy, radiotherapy, surgery, immunotherapy, etc.);<br>2) Stage IVb patients with metastatic parenchymal organs (such as liver, bone, lung, brain, adrenal, etc.) at the time of initial diagnosis;<br>3) Patients with a history of thoracic radiation therapy;<br>4) Multiple primary esophageal cancers;<br>5) Known or suspected allergy or hypersensitivity to monoclonal antibodies, any ingredients of toripalimab, and the chemotherapeutic drugs paclitaxel or cisplatin;<br>6) Female patients who are pregnant or lactating;<br>7) Inability to provide informed consent due to psychological, familial, social and other factors;<br>8) Patients with esophageal mediastinal fistula and/or esophageal tracheal fistula before treatment;<br>9) Presence of CTC grade ≥ 2 peripheral neuropathy;<br>10) A history of malignancies other than esophageal cancer before enrollment, excluding non-melanoma skin cancer, in situ cervical cancer, or cured early prostate cancer;<br>11) Patients who cannot tolerate chemoradiotherapy due to severe cardiac, lung, liver or kidney dysfunction, or hematopoietic disease or cachexia;<br>12) A history of diabetes for more than 10 years and poorly controlled blood glucose levels;<br>13) Active autoimmune diseases, a history of autoimmune diseases (including but not limited to these diseases or syndromes, such as colitis, hepatitis, hyperthyroidism), a history of immunodeficiency (including a positive HIV test result), or other acquired or congenital immunodeficiency diseases, a history of organ transplantation or allogeneic bone marrow transplantation;<br>14) A history of interstitial lung disease or non-infectious pneumonia<br>15) Presence of active hepatitis B (HBV DNA ≥ 2000 IU/mL or 104 copies/mL), hepatitis C (positive for hepatitis C antibody, and HCV-RNA levels higher than the lower limit of the assay);<br>16) Any unstable condition or condition that may compromise patients' safety and compliance. |

| | |
|---|---|
| **Duration of Trial** | Estimated enrollment time of the first subject：July 2019<br>Estimated enrollment time of the last subject：November 2020<br>Estimated end time of the study: November 2022 |
| **Therapeutic Regimen** | Concurrent chemoradiotherapy + Immunotherapy:<br>● Paclitaxel: 50 mg/m2, ivdrip, d1, 8, 15, 22, 29<br>● Cisplatin: 25 mg/m2, ivdrip, d1, 8, 15, 22, 29<br>● Toripalimab: 240 mg, ivdrip, d1,22<br>● Radiation therapy: intensity-modulated radiation therapy (IMRT), 50.4Gy/28F, five times a week<br>Immunomaintenance therapy:<br>● Toripalimab 240mg, ivdrip, repeated every 3 weeks for up to 1 year (a total of 17 cycles) or until disease progression, illness, unacceptable toxicity, investigator's decision, or patient withdrawal of consent. |
| **Sample Size and Statistical Methods** | ● All enrolled patients will be included in the analysis regardless of whether they complete the treatment protocol. Data analyses will be performed according to the "Intention to treat" principle.<br>● The primary endpoint of this study is cCR rate. A cCR rate of 40% can be expected based on historical data for definitive CRT in ESCC, and thus it is hypothesized that the cCR rate will increase to 64% for patients receiving combination of toripalimab and chemoradiotherapy. Assuming a two-sided alpha of 0.05 and a beta of 0.15, the sample size should be 37 patients. For a presumed deletion rate of 12%, 42 patients will be enrolled in this study.<br>● cCR, DOR, and safety will be analyzed by descriptive methods. OS and PFS will be calculated by Kaplan-Meier method and log-rank test will be performed. $P<0.05$ is considered statistically significant. |
| **Version Number** | 1.0 |

# 2. Summary

Definitive chemoradiotherapy (CRT) is the standard treatment option for unresectable esophageal cancer (EC). However, as high as 40% to 50% of EC patients experienced locoregional recurrence after definitive CRT. Immunotherapy targeting the PD-1/PD-L1 checkpoints has demonstrated promising activity in advanced EC. Preclinical studies have shown that radiotherapy can induce up-regulation of PD-L1 expression in esophageal cancer tissues, leading to immune escape of tumor cells, and the combination of PD-1/PD-L1 inhibitors and radiotherapy could play a synergistic anti-tumor effect. Therefore, PD-1/PD-L1 inhibitors combined with concurrent CRT may further improve the efficacy and prolong the survival, becoming a new approach for the treatment of EC. In view of the above problems, we aimed to conduct a prospective, single-arm, phase-II clinical trial to evaluate the efficacy, safety, and potential biomarkers of the combination of toripalimab and concurrent CRT for patients with locally advanced ESCC. The study plans to enroll 42 patients with unresectable locally advanced ESCC. Toripalimab and chemotherapy are performed simultaneously with radiotherapy. Intensity-modulated radiation therapy (IMRT) technique is used for a total dose of 50.4 Gy in 28 fractions. After the cpmpletion of CRT, patients will receive an additional 15 cycles of toripalimab (240 mg) every 3 weeks, for up to 1 year (a total of 17 cycles) or until disease progression, illness, unacceptable toxicity, investigator's decision, or patient withdrawal of consent.

# 3. Background

Esophageal cancer (EC) is the seventh most common and sixth most deadly cancer worldwide in 2018. More than 50% occur in China[1]. In China, nearly 375,000 EC patients died every year, and over 90% of Chinese EC patients are esophageal squamous cell carcinoma (ESCC). Besides, EC is the third-most common cause of cancer-related death in China. The overall prognosis remains poor, with local recurrence and distant metastasis being the major features of failure, which seriously threatens human life and health[2]. Radiotherapy is one of the main treatment methods for EC. Compared with radiotherapy alone, concurrent CRT can significantly improve the local control rate and survival rate, and has become the standard treatment for locally advanced esophageal cancer and an alternative treatment for patients who refuse surgery. However, the efficacy remains unsatisfactory, and the 5-year survival rates ranging from 15% to 25%[3-5]. The major failure types of EC after CRT are local recurrence and distant metastasis, in which the local recurrence rate is 45%-50%, and up to 75% of patients eventually develop distant metastasis[3-6]. Therefore, how to improve the poor prognosis of patients with locally advanced EC is an urgent clinical problem to be solved.

In recent years, programmed cell death-1 (PD-1) and programmed cell death-ligand 1 (PD-L1) have been the research hotspot in tumor immunotherapy. PD-1 belongs to the B7/CD28 family and is a type I transmembrane protein composed of 288 amino acids. It is mainly expressed on the membrane surface of activated T cells, B cells, NK cells, dendritic cells and monocytes. As the main ligand of PD-1, PD-L1 can be expressed in all antigen presenting cells, vascular endothelial cells, testis, placenta, cornea and other cells[7]. A number of studies have confirmed that PD-L1 is highly expressed in a variety of malignant tumor tissues, including EC[8]. Binding of PD-L1 with PD-1 can significantly inhibit the function of cytotoxic T cell, induce the production of regulatory T cells and regulate the secretion and expression of cytokines, leading to immune escape of tumor cells. Several studies have shown that PD-L1 expression is related to the clinicopathological characteristics of EC, such as tumor invasion depth, lymph node metastasis, pathological differentiation degree, and TNM stage[9,10]. According to the study reported by Yagi et al, the expression of PD-L1 was an independent prognostic factor for poor overall survival (OS) and disease-free survival (DFS) in EC patients[11].

Since the PD-1/PD-L1 signaling pathway is closely associated with tumor immune escape, immune checkpoint inhibitors targeting the PD-1/PD-L1 pathway provide a new therapeutic option for patients with EC. PD-1 /PD-L1 antibody can specifically bind to PD-1 or PD-L1, block the PD-1/PD-L1 signaling pathway, and enable T cells to resume the immune response to tumors. Currently, it has shown good efficacy and safety in advanced EC[12-16]. The multicenter, phase-III ATTRACTION-2 trial, which has included 493 patients with gastroesophageal junction carcinoma who had failed second-line therapy, has demonstrated that nivolumab can significantly increase the median OS of patients with gastroesophageal junction cancer compared to placebo (5.3 months vs. 4.1 months, $P<0.001$)[12]. The multicenter, phase-II study reported by Kudo et al. showed an objective remission rate (ORR) of 17%, a disease control rate (DCR) of 67%, and a median OS of 10.8 months in ESCC patients who received nivolumab alone as second-line treatment, in which only 26% patients with grade 3-4 adverse effects and no deaths related to treatment were

observed[13]. Recently, KEYNOTE-028 study reported an ORR of 30% in advanced EC patients who received pembrolizumab with positive expression of PD-L1 and failure of first-line chemotherapy, and only 17% patients with grade 3 adverse effects were observed[15]. Besides, JS001 (toripalimab) study reported an ORR of 27.1% in ESCC patients who received toripalimab alone as second-line treatment, and only 15.3% patients with grade 3 adverse effects were observed, which showed good security[17]. Notably, only partial EC patients achieved a permanent effect and the ORR was unfavorable ranging from 12% to 30%. Therefore, immunotherapy combined with radiotherapy, chemotherapy, and surgery, so as to further improve the efficacy of comprehensive treatment, might be a better regimen for EC.

It was found that chemotherapeutic drugs play an anti-tumor role by regulating the immune system of the body, causing the immunogenic death of tumor cells, increasing the antigenic cross-presentation ability of dendritic cells, activating the anti-tumor immune effect of the body, reducing the immunosuppression induced by myeloid inhibitory cells, increasing the proportion of cytotoxic lymphocytes and regulatory T cells, reducing the inhibitory effect of regulatory T cells on immunity, blocking the STAT6 pathway, down-regulating the expression of PD-L2 in dendritic cells and tumor cells, increasing T cell activity and tumor cell recognition. Besides, high expression of PD-L1 is associated with tumor invasion and chemotherapy resistance[18]. Therefore, the combination of immune checkpoint inhibitors and chemotherapy may play a synergistic antitumor effect. Several clinical trials in lung cancer have demonstrated that PD-1 /PD-L1 antibodies in combination with chemotherapy agents are superior to chemotherapy alone[19,20]. Currently, phase III studies are ongoing to investigate the efficacy of immunotherapy (Nivolumab, Pembrolizumab, and Toripalimab) combined with chemotherapy as first-line treatment for advanced EC patients.

Preclinical studies have shown that radiotherapy can induce tumor-infiltrating lymphocyte aggregation and up-regulation of PD-L1 expression in tumor tissues in a dose-dependent manner, resulting in tumor immune escape and inhibition of antitumor efficacy[21,22]. Lim et al. compared the changes of PD-L1 expression level in tissue specimens of EC before and after neoadjuvant CRT, and confirmed the up-regulation of PD-L1 expression induced by radiotherapy in human body[23]. Animal model studies have shown that radiotherapy combined with PD-L1 antibody can regulate the tumor microenvironment, reduce the accumulation of myeloid suppressor cells, remove the immunosuppressive effect induced by it, and simultaneously enhance the secretion of T cell-derived anti-tumor cytokines, increase the lethality to tumor cells, and enhance the efficacy of radiotherapy[24]. Dovedi et al. further found that for animal models using conventional segmented radiotherapy, the simultaneous application of PD-L1 antibody and radiotherapy had better efficacy than sequential application because of the short up-regulation period of PD-L1 expression after radiotherapy[25]. Therefore, the simultaneous combination of PD-1/PD-L1 antibody with CRT may further play a synergistic anti-tumor effect, increase the radiosensitivity of EC cells, thus improving the efficacy and prolonging the survival period, becoming a new method for the treatment of EC.

In this context, this study intends to carry out a prospective, single-arm, phase-II clinical study on the combination of PD-1 antibody (toripalimab) and definitive CRT in the treatment of locally advanced ESCC. This study aims to evaluate the efficacy and safety of the combination of toripalimab and definitive CRT in locally advanced ESCC, so as to further

optimize the comprehensive treatment modality.

# 4. Objective

To evaluate the efficacy, safety, and potential biomarkers of the combination of toripalimab and definitive CRT in locally advanced ESCC, and to provide a reliable theoretical basis for optimizing the comprehensive treatment mode of ESCC.

# 5. Plan

## 5.1 Patient Selection

### 5.1.1 Inclusion criteria

1) Histologically confirmed squamous cell carcinoma of the esophagus;
2) Not suitable for surgery (either for medical reasons or patient's choice);
3) No prior cancer therapy;
4) Estimated life expectancy >6 months;
5) Age at diagnosis being 18-70 years;
6) The function of important organs meets the following requirements: a. white blood cell count (WBC) ⩾ 4.0×109/L, absolute neutrophil count (ANC) ⩾ 1.5×109/L; b. platelets ⩾ 100×109/L; c. hemoglobin ⩾ 9g/dL; d. serum albumin ⩾ 2.8g/dL; e. total bilirubin ⩽ 1.5×ULN, ALT, AST and/or AKP ⩽ 2.5×ULN; f. serum creatinine ⩽ 1.5×ULN or creatinine clearance rate >60 mL/min; no serious organic diseases;
7) PS score ≤2;
8) Ability to understand the study and sign informed consent.

### 5.1.2 Exclusion criteria

1) Patients who have been treated previously with anti-tumor therapy (including chemotherapy, radiotherapy, surgery, immunotherapy, etc.);
2) Stage IVb patients with metastatic parenchymal organs (such as liver, bone, lung, brain, adrenal gland, etc.) at the time of initial diagnosis;
3) A history of radiation therapy to the chest;
4) Multiple primary esophageal cancers;

5) Known or suspected allergy or hypersensitivity to monoclonal antibodies, any ingredients of Toripalimab, and the chemotherapeutic drugs paclitaxel or cisplatin;

6) Female patients who are pregnant or lactating;

7) Inability to provide informed consent due to psychological, familial, social and other factors;

8) Patients with esophageal mediastinal fistula and/or esophageal tracheal fistula prior to treatment;

9) Presence of CTC grade ≥ 2 peripheral neuropathy;

10) A history of malignancies other than esophageal cancer before enrollment, excluding non-melanoma skin cancer, in situ cervical cancer, or cured early prostate cancer;

11) Patients who cannot tolerate chemoradiotherapy or surgery due to severe cardiac, lung, liver or kidney dysfunction, or hematopoietic disease or cachexia;

12) A history of diabetes for more than 10 years and poorly controlled blood glucose levels;

13) Active autoimmune diseases, a history of autoimmune diseases (including but not limited to these diseases or syndromes, such as colitis, hepatitis, hyperthyroidism), a history of immunodeficiency (including a positive HIV test result), or other acquired or congenital immunodeficiency diseases, a history of organ transplantation or allogeneic bone marrow transplantation;

14) A history of interstitial lung disease or non-infectious pneumonia;

15) Presence of active hepatitis B (HBV DNA ≥ 2000 IU/mL or 104 copies/mL), hepatitis C (positive for hepatitis C antibody, and HCV-RNA levels higher than the lower limit of the assay);

16) Any unstable condition or condition that may compromise patient safety and compliance.

### 5.1.3 Withdrawal criteria

1) Patients themselves or their legal representatives requested withdraw from the study;

2) Continuation of the treatment protocol detrimental to patients' health;

3) Esophageal perforation, severe lung/mediastinal infection, bleeding, myocardial infarction, heart failure, severe arrhythmia and other complications occurred during induction chemotherapy;

4) Patients with distant metastasis during CRT;

5) Pregnancy;

6) All patients who dropped out should be followed up according to the study protocol, and the follow-up results should be recorded, unless the patients withdrew the informed consent and refused to accept the study follow-up.

### 5.1.4 Eliminate criteria

1) Violation of the requirements of the research protocol;
2) Poor quality of data recording, incomplete and inaccurate data.

## 5.2 Examinations and screening of patients

### 5.2.1 Examinations

1) Complete medical history and systemic physical examinations (symptoms, signs, body weight loss and function score). It is generally required to be completed within 7-10 days before recruitment.
2) Pre-treatment examinations:

- Blood routine, blood type, urine routine, stool routine, biochemical routine, CB4, thyroid function, plasma cortisol;
- Hepatitis virus examination. If HBsAg is positive, HBV-RNA should be tested; If HCV-Ab is positive, HCV-RNA should be tested;
- Electrocardiogram;
- Ultrasonic cardiogram (UCG);
- Lung function tests;
- Histopathological/cytological diagnosis: pathological examination will be done based on the tissues from endoscopic biopsy;
- Esophageal barium swallowing;
- Chest and abdominal CT (with contrast);
- Esophagogastroduodenoscopy (EGD), with endoscopic ultrasound (EUS);
- Cervical ultrasonography;
- Electronic bronchoscopy or endobronchial ultrasound if necessary, to confirm the involvement of trachea and/or bronchus;
- Positron emission tomography–computed tomography (PET-CT);
- Quality of life questionnaire;
- Nutritional risk screening.

### 5.2.2 Screening of patients

Patients will be fully informed about the nature of the study before conducting research related tests. All potential patients will be screened according to the above-mentioned criteria, and those who meet the inclusion criteria and agree to sign informed consent forms will be recruited into the study.

## 5.3 Treatment Plan

### 5.3.1 Overall Design

This study, a prospective, single-center, single-arm, phase-II clinical trial, intends to enroll 42 patients with locally inoperable advanced ESCC. PD-1 antibody (toripalimab)/paclitaxel/cisplatin chemotherapy is concurrent with radiotherapy. After the end of CRT, PD-1 antibody monotherapy is continued until the disease progresses or clinical benefits could not be continued, and the longest duration of medication is 1 year.

### 5.3.2 Therapeutic Regimen

1) Chemotherapeutic drugs are administered on the first day of radiotherapy.

2) The dosing window is ±3 days from the planned dosing date (based on the first dosing date). If the dosing window period is exceeded, the dosing will be regarded as delayed, and the subsequent dosing date will be recalculated according to the actual dosing date of the last dosing. During the combination administration, if the delay is expected to exceed 2 weeks due to toxicity of chemotherapy, only toripalimab will be given until the toxicity is restored to the chemotherapy administration standard, and then the combination administration will be resumed. The maximum allowed continuous suspension of chemotherapy is 2 weeks, and the chemotherapy will be terminated after 2 weeks. If the delay is expected to exceed 2 weeks due to toxicity of toripalimab, only chemotherapy will be given until the toxicity is restored to the toripalimab administration standard, and then the combination administration will be resumed. The maximum allowed continuous suspension of toripalimab is 12 weeks, and the toripalimab will be terminated after 12 weeks. If a delay is required for toxicity reasons (not clear which drug is involved), all three drugs will need to be delayed at the same time if it is expected to return to re-dosing criteria within 2 weeks.

3) Protocols of drugs delivery

| | Toripalimab | Paclitaxel | Cisplatin |
|---|---|---|---|
| Dosage and route of administration | 240 mg ivdrip | 50 mg/m2 ivdrip | 25 mg/m2 ivdrip |
| Transfusion speed | ≥30 min， ≤60 min， including saline flush | 120 min | 120 min |
| Pretreatment | Not required | Required | Required |
| Administration time | D1,22 | D1, 8, 15, 22, 29 | D1, 8, 15, 22, 29 |

| | |
|---|---|
| Administration sequence | After toripalimab infusion, paclitaxel and cisplatin will be given at least 60 minutes interval. |
| Maintain dosing frequency | Toripalimab is administered every 3 weeks after CRT, with a maximum duration of 1 year. |

### 5.3.3 Management of toripalimab infusion reaction

| CTCAE | Symptoms | Treatment | Toripalimab |
|---|---|---|---|
| Grade 1 | Mild | Bedside observation and close monitoring until recovery.<br>Prophylaxis is recommended for future infusion: diphenhydramine 50mg, or equivalent and/or acetaminophen 325-1000mg, at least 30 minutes before administration of pd-1 antibody. | Continue |
| Grade 2 | Moderate<br><br>Requiring treatment or suspension.<br>Rapid remission after treatment (e.g., antihistamines, nonsteroidal anti-inflammatory drugs, anesthetics, bronchodilators, intravenous fluids, etc.) | Intravenous saline, diphenhydramine 50mg or equivalent and/or paracetamol 325-1000mg.<br>Bedside observation and close monitoring until recovery.<br>Corticosteroids or bronchodilators may be considered according to clinical needs.<br>Original medical records to study drug infusion volume.<br>Prophylaxis is recommended for future infusion: diphenhydramine 50mg, or equivalent and/or acetaminophen 325-1000mg, at least 30 minutes before administration of pd-1 antibody. When necessary, cortisol (equivalent to a 25mg dose of hydrocortisone). | Pause.<br>50% initial infusion rate when retaking after symptom resolution.<br>If there are no complications within 30 minutes, the infusion rate can be increased to 100%.<br>Monitor closely, and if symptoms recur, no PD-1 antibody is administered |
| Grade ⩾ 3 | Severe<br><br>No immediate response to treatment and/or suspension;<br>Or recurrence of symptoms after remission;<br>Sequelae that require | Stop the infusion of PD-1 antibody immediately; Start an i.v. drip of saline.<br>Bronchodilators are recommended by subcutaneous injection of 0.2-1mg of 1:1000 epinephrine solution, or 0.1-0.25mg of 1:10,000 epinephrine solution slowly intravenously, if necessary, and/or diphenhydramine 50mg plus methylprednisolone 100mg or equivalent intravenously.<br>Follow institute guidelines for treating | Terminate |

| | | | |
|---|---|---|---|
| | hospitalization. | anaphylaxis.<br>Bedside observation and close monitoring until recovery. | |

### 5.3.4 Pretreatment before chemotherapy

1) Prophylactic antiemetic therapy:

Acute and delayed vomiting induced by cisplatin must be prevented. Aprepitant + 5-$HT_3$ antagonist + dexamethasone is recommended within 1 hour prior to chemotherapy.

2) Allergy prevention:

Paclitaxel is pretreated with adrenocortical hormones (e.g., Dexamethasone), diphenhydramine, and H2-receptor antagonists (e.g., Cimetidine or Ranitidine).

3) Adjustment for allergy:

| Allergic symptoms | Treatment |
|---|---|
| Grade1: Local skin reactions such as mild itching, flushing and rashes | -Reduce the infusion rate until the symptoms disappear.<br>-Observe and monitor patients at the wards.<br>-Then continue dripping all paclitaxel at the original speed. |
| Grade2: Any symptoms not listed above (mild symptoms) or below (severe symptoms), such as systemic pruritus, flushing, rash, dyspnea and hypotension with systolic blood pressure >80 mm Hg | -Stop dripping paclitaxel.<br>-Administer DPH 50 mg IV with or without DXM 10 mg IV until the symptoms disappear.<br>-Then continue dripping paclitaxel at a lower speed and gradually to the original speed. |
| Grade3/4:Any severe symptoms such as bronchospasm, systemic rubella, systolic blood pressure ≤80mmHg and vascular edema | -Stop dripping paclitaxel.<br>-Administer DPH 50 mg IV with or without DXM 10 mg IV, and administer adrenaline, if necessary, until the symptoms disappear.<br>-Patients will withdraw from the study |
| Notes:Patients who has severe allergic symptoms will withdraw from the study, and the followed treatment will be decided by researchers. | |

### 5.3.5 Principles for the adjustment of dosage of drugs

**1) Toripalimab**

Adverse effect (AE) related to toripalimab may be associated to the immune system, which may occur at the first administration or a few months after the last administration. When symptoms listed below occur, the administration of toripalimab should be suspended or terminated if necessary. The resumption of regime is no longer than 12 weeks, otherwise it should be terminated.

| Immune-associated AE for interruption | Conditions for resumption |
|---|---|
| Diarrhea / Colitis | |
| Grade 2/3 | Recovery to grade 0-1 and corticosteroid reduced to less of prednisone 10 mg or its equivalent |
| Grade 4 | Termination |
| Hepatic dysfunction | |
| Grade 2 | Recovery to grade 0-1 and corticosteroid reduced to less of prednisone 10 mg or its equivalent |
| Grade 3/4 | Termination |
| Hyperthyroidism | |
| Grade 3 | Recovery to grade 0-1 and corticosteroid reduced to less of prednisone 10 mg or its equivalent |
| Grade 4 | Termination |
| Hypothyroidism | The start of thyroid hormone replacement therapy |
| Pneumonia | |
| Grade 2 | Recovery to grade 0-1 and corticosteroid reduced to less of prednisone 10 mg or its equivalent |
| Grade 3/4 | Termination |
| hypophysis | |
| Grade 2/3 | Recovery to grade 0-1 and the start of endocrine replacement therapy |
| Grade 4 | Termination |
| Newly diagnosed type I diabetes or grade 3/4 | Clinical and metabolic stabilization |

| | |
|---|---|
| hyperglycemia with β-cell failure | |
| Renal failure or nephritis | |
| Grade 2 | Recovery to grade 0-1 and corticosteroid reduced to less of prednisone 10 mg or its equivalent |
| Grade 3/4 | Termination |
| Transfusion reaction | |
| Grade 2 | Disappearance of symptoms |
| Grade 3/4 | Termination |
| Other AE related to toripalimab | |
| Grade 3 | Recovery to grade 0-1 and corticosteroid reduced to less of prednisone 10 mg or its equivalent |
| Grade 4 | Termination |

Notes: Regime should be terminated for any recurrence of grade 3 AE or any life-threatening event. For patients with liver metastasis and grade 2 elevation of AST or ALT at baseline, regime should be terminated if AST or ALT is more than 50% of the baseline and lasts for at least 1 week. Toripalimab can be suspended or terminated by researchers for patients with intolerable or persistent grade 2 AE. If persistent grade 2 adverse events do not return to grade 0-1 within 12 weeks of the last administration, toripalimab should be terminated.

**2) Paclitaxel/Cisplatin**

Highest dose of chemotherapy is given and adjusted according to the most severe toxicity. Patient will continue to receive reduced dose chemotherapy once dose is adjusted. The minimum dose should be selected if multiple toxicities occur. After two times of adjustment of dose, chemotherapy must be terminated if adjustment is necessary for the third time. Chemotherapy can only be delayed for up to 2 weeks, otherwise it should be terminated.

| AE | Paclitaxel | Cisplatin |
|---|---|---|
| Febrile neutropenia<br>Grade 4 neutropenia<br>Grade 4 thrombocytopenia | 20% reduction | 20% reduction |

| | | |
|---|---|---|
| Grade 1 renal toxicity<br>Grade 2/3 peripheral nerve toxicity<br>Grade ≥3 non-hematological toxicity | 20% reduction | 20% reduction |
| Grade ≥2 renal toxicity<br>Grade 4 peripheral nerve toxicity | Termination | Termination |

**Dose level of paclitaxel/cisplatin**

| Drugs | Level | Dose | Percentage |
|---|---|---|---|
| Paclitaxel | Highest | 50 $mg/m^2$ | 100% |
| | -1 | 40 $mg/m^2$ | 80% |
| | -2 | 30 $mg/m^2$ | 60% |
| Cisplatin | Highest | 25 $mg/m^2$ | 100% |
| | -1 | 20 $mg/m^2$ | 80% |
| | -2 | 15 $mg/m^2$ | 60% |

Notes: Evaluation criteria for acute toxicity of chemotherapy: CTCAE 4.0 toxicity evaluation criteria.

### 5.3.6 Radiotherapy scheme

1) During CT simulation, patients will be immobilized with a vacuum bag in the supine position with arms raised. Radiation will be delivered by intensity-modulated radiotherapy (IMRT) using 6-8 MV X-ray.

2) Gross tumor volume (GTV) is defined as the primary tumor and involved lymph nodes on CT and EUS. Clinical target volume (CTV) is defined as the primary tumor plus 3-cm proximal and distal margins and a radial margin of 0.5–1.0 cm, as well as the nodal GTV plus a 0.5-cm expansion. Planning target volume is determined by adding a 0.5-0.8 cm margin to the CTV.

3) Radiotherapy plan design: IMRT or Volumetric Modulated Arc Therapy (VMAT) technology, isocentric irradiation, and evaluation and optimization of treatment plan according to dose volume histogram, fault dose distribution, NTCP model. Planning evaluation criteria refer to QUANTEC standard (IJROPB, 2013).

4) Radiation dose: total dose is 50.4 Gy in 28 fractions, 5 times a week.

5) Organs at risk (OARs)

| Organs at risk | Dose limitation |
|---|---|
| Spinal cord | Dmax<45Gy |
| Lungs | V20 <30%, V5<65%, Dmean<17 Gy |
| Stomach | D1cc<54 Gy, V40<60% |
| Liver | V20 <30%, V30<20%, Dmean<23 Gy |
| Heart | Dmean<26Gy, V30<40%. Every effort should be made to keep the total heart dose to a minimum. |

### 5.3.7 Criteria for radiation-related toxicity

1) Continue radiotherapy if grade 3 toxicity is unrelated to radiotherapy, and the adjustment of chemotherapy is according to Table 4. Radiotherapy will be withheld if any grade 4 toxicity is observed.

2) When grade 3 radiation-related toxicity is observed, active symptomatic treatment will be administered and radiotherapy will be withheld until the toxicity has recovered to grade 2.

3) If any of the following toxicity is present, patients will be excluded from the treatment protocol: heavy hemorrhage, non-healing esophageal tracheal leakage, myocardial infarction, heart failure, severe arrhythmias, and severe radiation pneumonia with dyspnea.

### 5.3.8 Regular evaluation during CRT

Regular evaluation will consist of: routine blood tests, liver and kidney function once a week; esophageal barium x-ray examination after the patient received radiotherapy with 20 Gy and 40 Gy. All patients fill in the quality of life questionnaire-EORTC-QLQ-C30 version3 at the end of radiotherapy and chemotherapy. Adverse reactions, concomitant medication, and whether the study is terminated early need to be recorded.

### 5.3.9 Regular evaluation during maintenance

Routine blood tests and liver and kidney function are performed before each course of treatment. ECG and thyroid function are reviewed every 2 courses. Fill in quality of life questionnaire EORTC-QLQ-C30 version3 every 3 months. Adverse reactions, concomitant medication, and whether the study is terminated early need to be recorded.

### 5.3.10 Concomitant Therapy

1) During the administration period of this clinical study, the use of other anti-tumor drugs, immunotherapy and biotherapy that are not approved by this protocol shall be stopped.
2) Symptomatic medications can be administered, including prophylactic antiemetic drugs and G-CSF when blood drops. Prophylaxis with hematopoietic growth factor is permitted to avoid treatment interruption or delay. All symptomatic medications should be recorded and explained in detail on the CRF table.
3) Local use of corticosteroids is allowed, such as eye, nasal, intraarticular, inhaled, etc., and pretreatment of corticosteroids before chemotherapy is allowed.
4) Avoid alcoholic beverages during treatment.

## 5.4 Management of pseudo-progression

### 5.4.1 Definition

Some subjects may experience a temporary tumor outbreak in the first few months after initiation of immunotherapy, followed by a disease response, so subjects are allowed to continue with the original treatment regimen after the first onset of progressive disease (based on RECIST1.1 criteria).

A tumor outbreak can include any of the following: deterioration of the original target lesion; deterioration of the original non-target lesion; a new lesion appears.

### 5.4.2 Management

The investigator can decide whether to continue the study treatment based on the subjects' overall clinical condition, including physical condition, clinical symptoms and laboratory results. If the subjects are clinically stable, they can continue treatment and have their tumor evaluated again after an interval of at least 4 weeks (±7 days). According to iRECIST and RECIST 1.1 criteria, if PD is unconfirmed, the treatment should be continued. If PD is confirmed, treatment will be discontinued unless the investigator determines that the subject will continue to benefit clinically and allows the subject to continue treatment after PD is confirmed. For clinically unstable subjects, treatment should be discontinued after the first evaluation of PD, without the need for repeated imaging examination to confirm PD. For subjects who are first evaluated for PD, whether or not they continue to study therapy after the progression, the initial progression date assessed by the investigator will be used for all statistical analyses that include progression information.

Definition of clinical stability: no significant decrease in physical status and no significant worsening of tumor-related symptoms; no rapid disease progression; no advanced tumors at critical anatomical sites requiring urgent medical intervention (e.g. spinal cord compression). The criteria for PD:

| | Confirm PD (Any of the following) | Unconfirm PD (All of the following) |
|---|---|---|

| | | |
|---|---|---|
| Target lesion | The tumor load increased by ≥5 mm in absolute terms compared to the first progression. | The tumor load increased by <5 mm in absolute terms compared to the first progression. |
| Non-target lesion | Continuous progression of compared with initial progression (qualitative). | No clear progress compared to initial progress (qualitative). |
| New lesion | (1) Compared with the first progression, new lesions appeared.<br>(2) If a new lesion has appeared before, the new lesion increases, or other new lesions appear. | (1) Compared with the first progression, there were no other new lesions.<br>(2) If a new lesion has appeared before, the new lesion is stable or shrunken. |

## 5.5 Tumor and blood samples collection

### 5.5.1 Collection time

Baseline tumor biopsies are collected. Blood samples are collected before, during, and after CRT.

### 5.5.2 Processing and storage

A total of 4-6 mL of venous blood is collected at each time point and placed in the serum separation tube, which is placed in a cryogenic refrigerator.

## 5.6 Observing targets

### 5.6.1 Toxicity evaluation

Therapeutic toxicity is evaluated according to CTCAE 4.0 criteria.

### 5.6.2 Evaluation of short-term clinical efficacy after CRT

1) Evaluation time: 3 months after CRT (approximately the 18th week of treatment).
2) Evaluation measures: chest and abdomen enhanced CT, PET-CT, and EGD with biopsies.
3) Evaluation criteria: according to RECIST 1.1criteria.

Complete response (CR) is defined as the disappearance of lesion in esophagus by utilizing comprehensive methods: 1) no lesion, budding or ulceration identified by EGD; 2) negative pathologic findings by biopsies; 3) no evidence of local or distant recurrences in PET imaging, and a maximum standardized uptake value (SUVmax) in the primary region at a physiologic level or distributing in the esophagitis pattern.

Partial response (PR) is defined as at least a 30% decrease in the sum of diameters of target lesions, taking as reference the baseline sum diameters.

Progressive disease (PD) is defined as at least a 20% increase in the sum of

diameters of target lesions (an absolute increase of at least 5 mm), or the appearance of one or more new lesions.

Stable disease (SD) is defined as neither sufficient decrease to qualify for partial response nor sufficient increase to qualify for progressive disease.

Note: According to CT evaluation, the longest diameter of esophageal lesions is defined as the sum of the maximum diameters of the longitudinal axis and the horizontal axis, in which the maximum diameters of the horizontal axis are defined as the length of the maximum cross-section of the tumor minus the length of the central cavity on the same measurement line.

4) If there is any ambiguous finding, re-evaluation will be conducted within 6 weeks to determine the final response.

### 5.6.3 Endpoint evaluation

1) Primary endpoint: cCR.

2) Secondary endpoint: PFS、OS、DOR、Toxicity、Quality of life.

3) Exploratory endpoint: To investigate the impact of PD-L1 expression, immune cell infiltration in the tumor microenvironment, tumor mutational burden, genetic biomarkers, and dynamic ctDNA on clinical response and survival.

### 5.6.4 Follow-up

Patients will be followed up every 9 weeks for the first year, every 3 months for the second year, and then every 6 months thereafter. The follow-up contents include physical examination, serological examination (blood routine, biochemical routine, thyroid function, cortisol, etc.), esophageal barium swallowing, chest and abdomen enhanced CT, and PET-CT if necessary; in the first three years, esophagoscopy is performed every six months, and pathological biopsy will be performed if necessary.

## 5.7 Ethics

### 5.7.1 Informed consent

Before patients' recruitment, investigator should completely and comprehensively explain the objective of this study, the characteristics of drugs, and the potential toxicity and risk in the treatment, and allow the patients to be aware of their rights, risks and benefits. Informed consent form should be signed before recruitment and preserved in files as paper documentation.

### 5.7.2 Ethics and policy

This study will be conducted according to the Declaration of Helsinki (2000), Good Clinical Practice (GCP) published by CFDA and other relevant regulations. The

study must be approved by the Ethics Committee from leading center and each participating institution. Any amendments of the study protocol should be re-approved by the Ethics Committee during the study.

## 5.8 Quality guarantee

### 5.8.1 Requirements

In order to ensure that the trial can be carried out in strict accordance with the protocol, the clinical investigator should strictly follow the requirements of the GCP during the whole process of the clinical trial, and ensure that the trial procedure is standardized, the trial data is accurate and the study conclusions are reliable. Specific requirements are as follows:

1) Obtain informed consent signed by each subject or his/her agent;
2) Carefully fill in the case report form (CRF) as required;
3) Regularly follow-up;
4) Maintain complete laboratory examination records, clinical records, and subject's original medical records.

### 5.8.2 Data processing and preservation

1) Case Report Form (CRF): CRF should be filled in timely to assure information accuracy and prompt summary. CRF should generally not be altered. If there is indeed a mistake that need to be corrected, the investigator should sign at the site of alteration. CRF shall be filled out in duplicate and handed out to the sponsor and the investigator after the study. Data will be input into the database after being reviewed by the Clinical Research Associate (CRA), and all the content of CRF can no longer be amended afterwards.
2) Establishment of database: Once the CRF is received by the statisticians, the queries about the data should be answered by the investigator who filled in the CRF. The statisticians will then establish the database which is then reviewed and locked by the major investigators, sponsors, statisticians and CRA. Irrelevant persons have no access to the database. The database should be backed up.
3) Preservation of materials: According to the GCP, the documentations should be properly preserved by the investigators for more than 5 years.

## 5.9 Security measures

Results of studies conducted through this program may be published in medical journals, but we will keep patient information confidential as required by law. When necessary, government administrative departments and hospital Ethics Committees and their relevant personnel may consult the patient's data according to regulations.

## 5.10 Safety assessment

The information below is based on the results of non-clinical and clinical studies, as well as published data on similar drugs.

### 5.10.1 Safety plan

Various measures will be taken to ensure the safety of patient participated in this study, including strict inclusion and exclusion criteria and close monitoring. Study drug administration will be conducted in the presence of emergency medical facilities and staff trained in emergency surveillance and management. All adverse event (AE) and serious adverse event (SAE) will be recorded during the study.

1) Risks associated with toripalimab: The PD-L1/PD-1 pathway is involved in peripheral immune tolerance. Thus, such treatments may increase the risk of immune-mediated AE, particularly inducing or exacerbating autoimmune diseases. Potential immune-mediated AE have been observed in ongoing clinical studies of the safety and efficacy of toripalimab injection (JS001) in solid tumors, including interstitial lung disease, hypothyroidism and hyperthyroidism, liver dysfunction, pancreatitis, hyperglycemia, and adrenal insufficiency. For more details on clinical safety, please see the toripalimab injection (JS001) investigator's manual.

2) AE monitoring: This study will assess safety by monitoring all serious and non-serious AE (defined and graded according to the NCI CTCAE version 4.0 standard). Patients will be assessed for safety (including laboratory values) based on the duration of their visit. Laboratory values must be reviewed before each infusion. General safety assessments include a series of interphase histories, physical examinations, and specific laboratory studies, including serum chemistry and blood count. Patients will be closely monitored for signs and symptoms of autoimmune diseases and infections during the study. Reporting of all SAE will be expedited. Patients will be followed for safety for 60 days after the last dose of the study drug. After completion of study or exit to remain study treatment-related AE in patients with follow-up, ease to baseline levels, or until the researchers reckon the event has been stable, or start a new anti-cancer treatment, patients are lost to follow-up, or withdraw consent, or have been confirmed in patients' treatment or to participate in the study is not the cause of AE.

### 5.10.2 Safety parameters and definitions

The safety assessment includes monitoring and recording AE, including SAE, performing protocol-designated safety laboratory assessments, measuring protocol-designated vital signs, and performing other protocol-designated tests that are critical to the safety assessment of the study.

1) AE: An adverse event is any adverse medical event, regardless of causality,

that occurs in a clinical study subject receiving a drug product, according to the ICH Guideline for The Quality Management of Clinical Trials. Therefore, adverse events can be any of the following:

- Any adverse and unexpected signs (including abnormal laboratory findings), symptoms or illnesses associated with the time of drug use, whether or not they are believed to be related to the drug;
- Any new disease or exacerbation of an existing disease (an increase in the characteristic, frequency or severity);
- The condition is intermittent and recurrent (e.g.headache) and does not exist at baseline;
- Deterioration of laboratory values or other clinical findings (e.g. ECG, X-ray);
- AE associated with protocol-required interventions, including those that occurred before the study treatment was assigned (e.g. invasive procedures during screening, such as tissue biopsies).

2) SAE: A serious adverse event is an adverse event that meets any of the following criteria:

- Results in death;
- Is life-threatening (defined as when a subject is in danger of death at the time of the event);
- Requires hospitalization or prolonged hospital stay;
- Results in persistent or significant disability/incapacity;
- Leads to congenital anomalies or birth defects;
- Other significant medical events requiring intervention to prevent permanent injuries or damages.

### 5.10.3 Collect AE information

AE information should be solicited using a consistent, non-guided inquiry method at all patient evaluation time points. Example of a non-leading question: "How are you feeling since your last clinic visit?" "Have you had any new health problems or changes in your health since your last departure?"

### 5.10.4 Severity judgement of AE

Refer to the CTCAE Version 4 classification criteria for adverse drug reactions. The following criteria can be used as references if unlisted adverse reactions occur:

Grade I: Mild, asymptomatic or mild symptoms; clinical or laboratory test abnormality only; treatment not indicated.

Grade II: Moderate; minimal, local or non-invasive intervention required; limited age-appropriate instrumental activities of daily living (e.g., cooking, shopping, using the telephone and counting money, etc.).

Grade III: Severe or medically significant but not immediately life-threatening; leading to hospitalization or prolongation of hospitalization; leading to disabling; limiting self-care activities of daily living, but not bedridden. Self-care ADL: refers to bathing, dressing and undressing, feeding self, using the toilet, taking medications, and not bedridden.

Grade IV: Life-threatening consequences; urgent intervention indicated.

Grade V: Death related to AEs.

### 5.10.5 Causality judgement of AE

The investigator should determine whether an adverse event is related to the investigational drug based on the knowledge of patient, the context in which the event occurred, and an assessment of any possible alternative causes, and state "related" or "unrelated" accordingly. The following guidance should be considered:

- Event occur in a plausible time relationship to drug administration;
- The course of events, with special consideration of the effects of dose reduction, discontinuation of the study drug, or re-use of the study drug, if applicable;
- Known associations of events with the study drug or similar treatment;
- Known associations of events with study diseases;
- Patients with risk factors or use of concomitant medications known to increase event rates;
- Non-therapeutic factors known to be associated with the occurrence of the event;
- Patients receiving combination therapy are separately assessed for causality between adverse events and the treatment prescribed by the protocol.

### 5.10.6 AE recording procedure

The investigator should document AE on the AE page of the CRF using the correct medical terminology/concepts. Avoid colloquialisms and abbreviations. In the EVENT column of the AE page of CRF, only one AE term can be recorded for an event.

### 5.10.7 AE reporting

All SAE and AE of particular concern will be evaluated in a timely manner and reported promptly to the appropriate regulatory authorities and Ethics Committees in accordance with applicable legal requirements.

## 5.11 Statistical analysis

### 5.11.1

Professional statisticians will undertake the task of statistical analysis and participate in the whole process from experimental design, implementation to analysis and summary. After the completion of the test protocol and CRF, they will formulate the statistical analysis plan, make necessary modifications during the test, and provide the statistical analysis report after the completion of data analysis. All enrolled patients will be included in the analysis regardless of whether they complete the treatment protocol. Data analysis will be performed according to the "Intention to treat" (ITT) principle.

### 5.11.2 Sample size determination

The primary endpoint of this study is cCR. A cCR rate of 40% can be expected based on historical data for definitive CRT in ESCC, and thus it is hypothesized that the cCR rate will increase to 64% for patients receiving combination of toripalimab and CRT. Assuming a two-sided alpha of 0.05 and a beta of 0.15, the sample size should be 37 patients. For a presumed deletion rate of 12%, 42 patients will be enrolled in this study.

### 5.11.3 Statistical methods

cCR, DOR, and safety analysis will be performed by descriptive methods; OS and PFS will be calculated by Kaplan-Meier method and log-rank test will be performed. DOR definition: the time between the first tumor evaluation for CR or PR and the first evaluation for PD or death for any cause; OS definition: from date of enrollment to date of death or last follow-up; PFS definition: from date of enrollment to date of disease progression or death. $P<0.05$ is considered statistically significant.

# 6. Route

Newly treated patient with locally advanced ESCC

N=42

↓

Concurrent chemoradiotherapy + immunotherapy

paclitaxel（50 $mg/m^2$, ivdrip, d1, 8, 15, 22, 29）

cisplatin（ 25 $mg/m^2$, ivdrip, d1, 8, 15, 22, 29 ）

toripalimab （ 240 mg, ivdrip, d1, 22）

↓

Maintenance therapy

Toripalimab（240mg, ivdrip, q3w）

Treatment until PD is confirmed

or no clinical benefit continues

Maximum duration of medication: 1 year

↓

Follow-up

Statistical analysis

# 7. References

1) Bray F, Ferlay J, Soerjomataram I, et al. Global cancer statistics 2018: GLOBOCAN estimates of incidence and mortality worldwide for 36 cancers in 185 countries. CA Cancer J Clin. 2018; 68: 394-424..

2) Chen W, Zheng R, Baade PD, et al. Cancer statistics in China, 2015. CA Cancer J Clin. 2016;66:115-132.

3) Lordick F, Mariette C, Haustermans K, et al. Oesophageal cancer: ESMO Clinical Practice Guidelines for diagnosis, treatment and follow-up. Ann Oncol 2016;27(suppl 5):v50-v57.

4) Cooper JS, Guo MD, Herskovic A, et al. Chemoradiotherapy of locally advanced esophageal cancer: long-term follow-up of a prospective randomized trial (RTOG 85-01). Radiation Therapy Oncology Group. JAMA 1999;281:1623-1627.

5) Minsky BD, Pajak TF, Ginsberg RJ, et al. INT 0123 (Radiation Therapy Oncology Group 94-05) phase III trial of combined-modality therapy for esophageal cancer: high-dose versus standard-dose radiation therapy. J Clin Oncol 2002;20:1167-1174.

6) Sudo K, Xiao L, Wadhwa R, et al. Importance of surveillance and success of salvage strategies after definitive chemoradiation in patients with esophageal cancer. J Clin Oncol. 2014; 32: 3400-3405.

7) Freeman GJ, Long AJ, Iwai Y, et al. Engagement of the PD-1 immunoinhibitory receptor by a novel B7 familymember leads to negative regulation of lymphocyte activation. J Exp Med. 2000; 192: 1027-1034.

8) Ohigashi Y, Sho M, Yamada Y, et al. Clinical significance of programmed death-1 ligand-1 and programmed death-1 ligand-2 expression in human esophageal cancer. Clin Cancer Res. 2005; 11: 2947-2953.

9) Jiang Y, Lo AWI, Wong A, et al. Prognostic significance of tumor-infiltrating immune cells and PD-L1 expression in esophageal squamous cell carcinoma. Oncotarget. 2017; 8: 30175-30189.

10) Hynes CF, Kwon DH, Vadlamudi C, et al. Programmed death ligand 1: a step toward immunoscore for esophageal cancer. Ann Thorac Surg. 2018; in press.

11) Yagi T, Baba Y, Ishimoto T, et al. PD-L1 expression, tumor-infiltrating lymphocytes, and clinical outcome in patients with surgically resected esophageal cancer. Ann Surg. 2017; in press.

12) Kang YK, Boku N, Satoh T, et al. Nivolumab in patients with advanced gastric or gastro-oesophageal junction cancer refractory to, or intolerant of, at least two previous chemotherapy regimens (ONO-4538-12, ATTRACTION-2): a randomised, double-blind, placebo-controlled, phase 3 trial. Lancet. 2017; 390: 2461-2471.

13) Kudo T, Hamamoto Y, Kato K, et al. Nivolumab treatment for oesophageal squamous-cell carcinoma: an open-label, multicentre, phase 2 trial. Lancet Oncol. 2017; 18: 631-639.

14) Janjigian YY, Bendell J, Calvo E, et al. CheckMate-032 study: efficacy and safety of nivolumab and nivolumab plus ipilimumab in patients with metastatic esophagogastric cancer. J Clin Oncol. 2018; in press.

15) Doi T, Piha-Paul SA, Jalal SI, et al. Safety and antitumor activity of the anti-programmed death-1 antibody pembrolizumab in patients with advanced esophageal carcinoma. J Clin Oncol. 2018; 36: 61-67.

16) Fuchs CS, Doi T, Jang RW, et al. Safety and efficacy of pembrolizumab monotherapy in patients with previously treated advanced gastric and gastroesophageal junction cancer: phase 2 clinical KEYNOTE-059 trial. JAMA Oncol. 2018; 4: e180013.

17) Wang FH, et al. Safety and antitumor activity of the anti-programmed death-1 antibody JS001 in patients with advanced esophageal carcinoma. ASCO, 2018; abstract 116.

18) Wang Z, Till B, Gao Q. Chemotherapeutic agent-mediated elimination of myeloid-derived suppressor cells. Oncoimmunology. 2017; 6: e1331807.

19) Paz-Ares L, Luft A, Vicente D, et al. Pembrolizumab plus chemotherapy for squamous non-small-cell lung cancer. N Engl J Med. 2018; 379: 2040-2051.

20) Gandhi L, Rodríguez-Abreu D, Gadgeel S, et al. Pembrolizumab plus chemotherapy in metastatic non-small-cell lung cancer. N Engl J Med. 2018; 378: 2078-2092.

21) Zhang W, Pang Q, Zhang X, et al. Programmed death-ligand 1 is prognostic factor in esophageal squamous cell carcinoma and is associated with epidermal growth factor receptor. Cancer Sci. 2017; 108: 590-597.

22) Teng F, Kong L, Meng X, et al. Radiotherapy combined with immune checkpoint blockade immunotherapy: achievements and challenges. Cancer Lett. 2015; 365: 23-29.

23) Lim SH, Hong M, Ahn S, et al. Changes in tumour expression of programmed death-ligand 1 after neoadjuvant concurrent chemoradiotherapy in patients with squamous oesophageal cancer. Eur J Cancer. 2016; 52 :1-9.

24) Deng L, Liang H, Burnette B, et al. Irradiation and anti-PD-L1 treatment synergistically promote antitumor immunity in mice. J Clin Invest. 2014; 124: 687-95.

25) Dovedi SJ, Adlard AL, Lipowska-Bhalla G, et al. Acquired resistance to fractionated radiotherapy can be overcome by concurrent PD-L1 blockade. Cancer Res. 2014; 74: 5458-5468.
