# Supplementary material for: Predictive role of ctDNA in esophageal squamous cell carcinoma receiving definitive chemoradiotherapy combined with toripalimab
Source: Nat Commun. 2024 Mar 1;15:1919. doi: 10.1038/s41467-024-46307-7 (PMC10907344; doi:10.1038/s41467-024-46307-7)
Supplement: Supplementary file 3 — Reporting Summary [file 41467_2024_46307_MOESM3_ESM.pdf]

nature portfolio

Corresponding author(s): Mian Xi
Last updated by author(s): Jan 16, 2024

# Reporting Summary

Nature Portfolio wishes to improve the reproducibility of the work that we publish. This form provides structure for consistency and transparency in reporting. For further information on Nature Portfolio policies, see our Editorial Policies and the Editorial Policy Checklist.

## Statistics

For all statistical analyses, confirm that the following items are present in the figure legend, table legend, main text, or Methods section.

| n/a | Confirmed | |
|---|---|---|
| ☐ | ☒ | The exact sample size (*n*) for each experimental group/condition, given as a discrete number and unit of measurement |
| ☐ | ☒ | A statement on whether measurements were taken from distinct samples or whether the same sample was measured repeatedly |
| ☐ | ☒ | The statistical test(s) used AND whether they are one- or two-sided<br>*Only common tests should be described solely by name; describe more complex techniques in the Methods section.* |
| ☐ | ☒ | A description of all covariates tested |
| ☒ | ☐ | A description of any assumptions or corrections, such as tests of normality and adjustment for multiple comparisons |
| ☐ | ☒ | A full description of the statistical parameters including central tendency (e.g. means) or other basic estimates (e.g. regression coefficient) AND variation (e.g. standard deviation) or associated estimates of uncertainty (e.g. confidence intervals) |
| ☐ | ☒ | For null hypothesis testing, the test statistic (e.g. $F$, $t$, $r$) with confidence intervals, effect sizes, degrees of freedom and $P$ value noted<br>*Give P values as exact values whenever suitable.* |
| ☒ | ☐ | For Bayesian analysis, information on the choice of priors and Markov chain Monte Carlo settings |
| ☐ | ☒ | For hierarchical and complex designs, identification of the appropriate level for tests and full reporting of outcomes |
| ☒ | ☐ | Estimates of effect sizes (e.g. Cohen's *d*, Pearson's *r*), indicating how they were calculated |

*Our web collection on statistics for biologists contains articles on many of the points above.*

## Software and code

Policy information about availability of computer code

| | |
|---|---|
| Data collection | No software was used |
| Data analysis | Oncoprints were applied to visualize multiple genetic alterations. The 'ComplexHeatmap' package provides the oncoPrint() function in R (V 3.5.3). The 'survival' package in R (V4.1.2) were used to calculate probabilities and to plot KM curves. The code is accessible at https://github.com/b123r45678/R_code.git. |

For manuscripts utilizing custom algorithms or software that are central to the research but not yet described in published literature, software must be made available to editors and reviewers. We strongly encourage code deposition in a community repository (e.g. GitHub). See the Nature Portfolio guidelines for submitting code & software for further information.

## Data

Policy information about availability of data

All manuscripts must include a data availability statement. This statement should provide the following information, where applicable:

- Accession codes, unique identifiers, or web links for publicly available datasets
- A description of any restrictions on data availability
- For clinical datasets or third party data, please ensure that the statement adheres to our policy

The raw sequence data used in this study are available in the Genome Sequence Archive (Genomics, Proteomics & Bioinformatics 2021) in National Genomics Data Center (Nucleic Acids Res 2022), China National Center for Bioinformation / Beijing Institute of Genomics, Chinese Academy of Sciences database under accession

code HRA005042 (https://ngdc.cncb.ac.cn/gsa-human). OncoKB database used in this study are publicly available via https://www.oncokb.org/. The remaining data including individual de-identified participant data are available within the Source Data file.

## Research involving human participants, their data, or biological material

Policy information about studies with human participants or human data. See also policy information about sex, gender (identity/presentation), and sexual orientation and race, ethnicity and racism.

| | |
|---|---|
| Reporting on sex and gender | This study recruited male and female patients without any prejudice. Gender was determined based on self-reporting way. |
| Reporting on race, ethnicity, or other socially relevant groupings | No socially relevant categorization variables were used in this study. All included patients were Asian. |
| Population characteristics | Forty-two patients who had locally advanced esophageal squamous cell carcinoma enrolled in the clinical trial EC-CRT-001 were included in this study. All 42 patients started and followed the protocol-defined intervention. The median age was 56 years (IQR, 53–63) and 39 (93%) of 42 patients had stage III or IVA disease. The detailed information of population characteristics was listed in the additional file - Table S1. |
| Recruitment | Forty-two patients from Sun Yat-sen University Cancer Center (Guangzhou, China) were recruited for this study between November 2019 to January 2021 based on the inclusion and exclusion criteria. This information was also described in Methods section. No potential self-selection bias or other biases. |
| Ethics oversight | The Institutional Review Board of Sun Yat-sen University Cancer Center approved this study (IRB NO. B2019-065-01). |

Note that full information on the approval of the study protocol must also be provided in the manuscript.

# Field-specific reporting

Please select the one below that is the best fit for your research. If you are not sure, read the appropriate sections before making your selection.

☒ Life sciences ☐ Behavioural & social sciences ☐ Ecological, evolutionary & environmental sciences

For a reference copy of the document with all sections, see nature.com/documents/nr-reporting-summary-flat.pdf

# Life sciences study design

All studies must disclose on these points even when the disclosure is negative.

| | |
|---|---|
| Sample size | Forty-two patients who had locally advanced esophageal squamous cell carcinoma enrolled in the clinical trial EC-CRT-001 were included in this study. In EC-CRT-001, the sample size was determined according to the following: based on a two-sided type-I error of 0.05 and a power of 85%, a total sample size of 37 patients would be required to demonstrate an improvement of 24% in the complete rate rate at 3 months after radiotherapy (from 40% in the historical study to 64% as assumed). Assuming a 12% dropout rate, the final sample size was estimated as 42. |
| Data exclusions | Five patients with tissue specimens that failed in quality control were excluded for analyses. All ctDNA sequencing data met the quality control criteria and well included for analyses. |
| Replication | Not applicable in this clinical trial setting. |
| Randomization | This study was the exploratory analysis of the single-arm phase II trial (EC-CRT-001). No randomization was required. |
| Blinding | EC-CRT-001 was an open-label phase II trial. No blinding was required. |

# Reporting for specific materials, systems and methods

We require information from authors about some types of materials, experimental systems and methods used in many studies. Here, indicate whether each material, system or method listed is relevant to your study. If you are not sure if a list item applies to your research, read the appropriate section before selecting a response.

## Materials & experimental systems

| n/a | Involved in the study |
|---|---|
| ☒ | ☐ Antibodies |
| ☒ | ☐ Eukaryotic cell lines |
| ☒ | ☐ Palaeontology and archaeology |
| ☒ | ☐ Animals and other organisms |
| ☐ | ☒ Clinical data |
| ☒ | ☐ Dual use research of concern |
| ☒ | ☐ Plants |

## Methods

| n/a | Involved in the study |
|---|---|
| ☒ | ☐ ChIP-seq |
| ☒ | ☐ Flow cytometry |
| ☒ | ☐ MRI-based neuroimaging |

# Clinical data

Policy information about clinical studies

All manuscripts should comply with the ICMJE guidelines for publication of clinical research and a completed CONSORT checklist must be included with all submissions.

Clinical trial registration: ClinicalTrials.gov Identifier: NCT04005170.

Study protocol: The full trail protocol can be accessed in our previous published paper (DOI: 10.1016/S1470-2045(23)00060-8)

Data collection: Patients were eligible if they were aged 18–70 years; had previously untreated, unresectable, histologically confirmed stage I–IVA oesophageal squamous cell carcinoma, according to the 8th TNM staging system of the American Joint Committee on Cancer; had an ECOG performance status of 0–2; had an estimated life expectancy of at least 6 months. Between Nov 12, 2019, and Jan 25, 2021, 42 patients from Sun Yat-Sen University Cancer center were enrolled. Genomic DNA samples from FFPE sections of pretreatment biopsies were captured.Peripheral blood samples were collected longitudinally before, during, and after CRT, whenever possible.

Outcomes: The primary outcome of this clinical trial was the clinical complete response rate. Tumor response was assessed according to the Response Evaluation Criteria in Solid Tumors (RECIST; version 1.1) using CT, PET, and esophagogastroduodenoscopy. Complete response determined by PET was defined when the SUVmax in the primary region was at a normal physiological level or when SUVmax was higher than normal but exhibited a uniform distribution and overlapped with the radiation field, indicating esophagitis. Secondary endpoints were overall survival time, defined as the time from enrolment to death or censored at the last follow-up, and progression-free survival time, defined as from enrolment to disease progression or death from any cause or censored at the last follow-up. Exploratory outcomes included associations of clinical response and survival with ctDNA status, genetic biomarkers, and TMB.

# Plants

Seed stocks: Not applicable in this clinical trial setting.

Novel plant genotypes: Not applicable in this clinical trial setting.

Authentication: Not applicable in this clinical trial setting.
